# Molecular Epidemiology and Control Strategies for BVDV: A Global Systematic Review From 2000 to 2025

**DOI:** 10.1155/vmi/6732453

**Published:** 2025-11-12

**Authors:** Eaftekhar Ahmed Rana, Jully Gogoi-Tiwari, Joshua Aleri, M. Asaduzzaman Prodhan, Syeda H. Akter, Henry Annandale, Subir Sarker, Sam Abraham, Jasim M. Uddin

**Affiliations:** ^1^School of Veterinary Medicine, Murdoch University, Murdoch, Western Australia 6150, Australia; ^2^Department of Microbiology and Veterinary Public Health, Chattogram Veterinary and Animal Sciences University, Khulshi, Chattogram 4225, Bangladesh; ^3^School of Veterinary Science, The University of Queensland, Gatton, Queensland 4343, Australia; ^4^Biomedical Sciences & Molecular Biology, College of Medicine and Dentistry, James Cook University, Townsville, Queensland 4811, Australia; ^5^Australian Institute of Tropical Health and Medicine, James Cook University, Townsville, Queensland 4811, Australia; ^6^Centre for Biosecurity and One Health, Harry Butler Institute, Murdoch University, Murdoch, Western Australia 6150, Australia

**Keywords:** BVDV, diagnostic approach, genetic diversity, host diversity, prevention and control, risk factors

## Abstract

Bovine viral diarrhea virus (BVDV) remains a significant and highly contagious pathogen that markedly impacts production and reproductive performances of different animals worldwide. This review represents the global epidemiology of BVDV, emphasizing its genetic diversity, prevalence, host range, associated risk factors, diagnostic advancements, and control strategies. A systematic electronic search was performed to retrieve relevant published articles. A total of 248 studies published over the past 26 years (from January 2000 to March 2025) across 69 countries were included. Data showed that BVDV-1 has been detected across all the continents and comprises 25 subgenotypes (1a-1x and Chinese ZM-95), of which the predominant subgenotypes are 1a, 1b, and 1c. Multiple subgenotypes, such as BVDV-1f, 1g, 1h, 1k, 1l, 1r, 1s, 1t, 1u, and 1x, were distinct and circulating in European countries. Additionally, five subgenotypes (2a–2e) of BVDV-2 have been identified, with BVDV-2a being the most frequently reported in different geographical locations. Notably, the emergence of HoBi-like pestivirus subgenotypes (BVDV-3a–3d) has been detected in Russia, Italy, Thailand, India, and Bangladesh. Overall, the high prevalence of BVDV has been reported in various European (2.9%–87.1%) and Asian countries (0.2%–89.49%). Although cattle are the primary host, BVDV infections have been documented across a wide range of domestic and wild species, including buffalo, sheep, goats, deer, bison, yak, camelids (camels, alpacas, and llamas), pigs, and wild boar. While Ag/Ab-ELISA remains a widely used diagnostic method, advanced techniques, such as RT-qPCR, CRISPR-Cas12a, RT-LAMP, and genome sequencing, are utilized for confirmatory identification and genotyping of BVDV. Introduction of persistently infected (PI) animals into herds, grazing on common pasture, animal movements, mixed farming practices, and unhygienic breeding practices were frequently documented as potential risk factors. Key measures for controlling and eradicating BVDV include culling of PI animals, prophylactic vaccination, and avoiding mixed farming practices.

## 1. Introduction

Bovine viral diarrhea virus (BVDV) is a globally distributed endemic pathogen causing serious infectious diseases in livestock species including cattle [1]. The virus affects multiple organ systems in susceptible hosts and causes prolonged immunosuppression, reduced productivity, and severe reproductive and neonatal losses [2, 3]. It also causes acute gastrointestinal and respiratory disorders, and nonvisible teratogenic abnormalities, mostly in calves [2, 3]. Thus, BVDV imposes a significant economic burden on cattle herds, causing reduced reproductive performance and increasing costs associated with disease management and control [4]. According to the International Committee on Virus Taxonomy (ICTV), BVDV is a member of the genus *Pestivirus*, under the family *Flaviviridae*. BVDV genome is a positive-sense, single-stranded RNA (ssRNA) of 11.3–13.0 kb that contains a single large open reading frame (ORF) encoding a polyprotein that is flanked by a 5′-NCR (noncoding region) and a 3′-NCR [5]. BVDV virions comprise four structural and eight nonstructural proteins [5, 6], which are essential for viral characterization and play fundamental roles in replication and pathogenesis [3, 5].

Based on the genomic makeup (5′UTR, N^pro^, and E genes), BVDV is classified into distinct species, namely, BVDV-1 (*Pestivirus bovis*), BVDV-2 (*Pestivirus tauri*), and HoBi-like virus or BVDV-3 (*Pestivirus brazilense*) as well as different genotypes/subgenotypes [5, 7, 8]. Notably, mutations in the E2, Npro, and 5′UTR genes play a critical role in the evolution and emergence of novel BVDV genotypes and subgenotypes [5, 6]. All BVDV species are capable of establishing persistent infection (PI) in susceptible hosts, when pregnant dams are infected between 40 and 120 days of gestation [3]. Based on their effects on susceptible bovine cell cultures, BVDV isolates are classified into cytopathic (Cp) and noncytopathic (Ncp) biotypes [3, 5]. The Cp biotype of BVDV causes detrimental mucosal disease (MD), while the Ncp biotype is associated with PI in cattle [9]. The clinical conditions of BVDV infection often depend on the biotypes (Ncp or Cp) that infect the host. MD results from a superinfection of an animal persistently infected with an Ncp virus by a Cp virus [2, 3]. Nevertheless, BVDV lacks strict host specificity, as evidenced by its ability to infect members of over 50 mammalian species [3]. It poses a significant threat not only to economically important food animals but also to a diverse spectrum of wildlife species, representing its broad host range and complex epidemiology [3]. However, PI animals are considered a potential source of BVDV infection and play an important role in the virus's infection ecology [1, 7]. Viruses shedding from PI animals through multiple routes, including feces, urine, semen, colostrum/milk, nasal, and ocular discharges, lead to the eventual spread within and between farm environments [3]. Moreover, once wildlife species become infected with BVDV, interspecies contact and constant shedding of the virus trigger the establishment of wildlife infection and subsequently transient carrier of BVDV [3]. The presence of PI animals, variations in herd management and biosecurity practices, and the wide host tropism of BVDV contribute to its complex transmission dynamics and facilitate spillover events between different hosts, posing significant challenges for effective control measures [1, 10]. Although a range of advanced diagnostic tools is available, field-applicable and highly sensitive molecular surveillance methods are essential for early detection and the successful implementation of BVDV eradication programs [3, 7]. Previous reviews have often been regional or limited in scope. To our knowledge, no comprehensive global synthesis from 2000 to 2025 exists that integrates genetic diversity, host range, diagnostic approaches, risk factors, and control measures. This review addresses this gap. It is worthwhile to mention that understanding these aspects is crucial for developing effective control and prevention strategies for BVDV. Therefore, this review synthesizes and integrates current information and knowledge of BVDV epidemiology, focusing on its genetic diversity, host range, diagnostic approaches, associated risk factors, and control strategies.

## 2. Materials and Methods

### 2.1. Search Strategy and Data Sources

**Figure 1:**
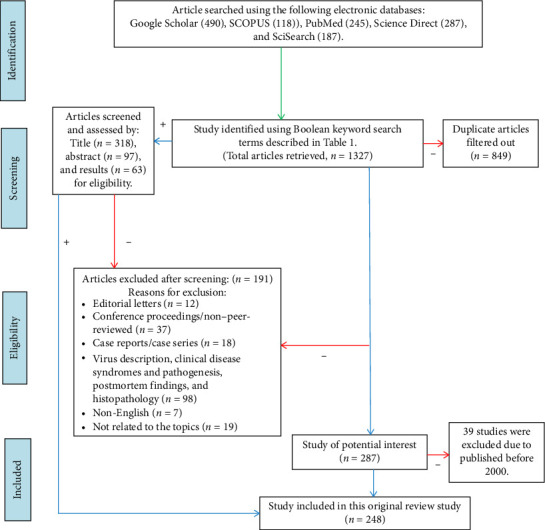
Systematic search for identification, screening, and selection of eligible peer-reviewed journal articles describing BVDV in different countries of the world. Here, the red arrow with a “–” indicates articles excluded from the current study, and the blue arrow with a “+” represents studies included in the systematic review.

For literature searching in the current systematic review, we followed the Preferred Reporting Items for Systematic Reviews and Meta-Analyses (PRISMA) protocol (Figure 1). An extensive literature search was commenced from January 1, 2025, to March 15, 2025, based on the discussion of authors (Eaftekhar Ahmed Rana, Jully Gogoi-Tiwari, and Jasim M. Uddin). Relevant literature and articles “Titles and Abstracts” were searched and screened using the following databases: Google Scholar, SCOPUS, PubMed, Science Direct, and Science Citation Index Expanded (SciSearch) for all relevant studies. All selected databases were accessed by using Boolean keyword search terms defined in Table 1. All articles were searched and screened for full text, and only original research and review articles were included. To ensure a systematic approach and improve the relevance of selected articles, only studies directly related to the targeted topics were included in this review (Table 1).

### 2.2. Eligibility and Study Selection Criteria

For the systematic review, specific eligibility and selection criteria were set to retrieve the relevant published articles. The searched articles were primarily considered for eligibility based on the following specific inclusion criteria: [i] article describing the genetic diversity of BVDV-1, BVDV-2, and HoBi-like pestivirus; study reporting genotypes and subgenotypes, reservoir and host range, diagnostic approach, risk factors, and prevention and control policy for BVDV; [ii] research studies describing BVDV infection in domestic and wildlife species, including but not limited to cattle, buffalo, sheep, goats, pigs (swine), deer, bison, yaks, camelids, and other wild animals; [iii] articles reported on any animal herds or production system, including both dairy and beef, as well as animal demographic and management-related risk factors associated with BVDV; [iv] article published between January 2000 and March 2025 focusing on selected topics from any countries of the world; [v] articles published in peer-reviewed journals in the English language; and [vi] available articles in full text. The exclusion criteria included all of the following studies or literature: [a] editorial letters, conference proceedings (non–peer-reviewed articles), case reports, case series, and commentaries; [b] studies not related to BVDV; [c] randomized/nonrandomized controlled trials, clinical trials using animal model, in vivo animal studies, and in vitro and cell-culture-based studies; [d] virus description, clinical symptoms and pathogenesis, and postmortem findings; [e] studies published before 2000; and [f] articles found in other languages. All article titles, abstracts, and results were independently screened in a blinded manner by the authors (Eaftekhar Ahmed Rana, and Jasim M. Uddin) based on the predefined inclusion and exclusion criteria. All duplications were omitted by the Mendeley reference manager, and discrepancies were solved through cross-screening, collective discussion, and consensus of the authors, such as Eaftekhar Ahmed Rana and Jasim M. Uddin. After that, the relevant checklist for systematic reviews was sequentially followed for the selection and exclusion of the published articles included in the current systematic review. The chronological procedures of the study are illustrated in the flow diagram in Figure 1.

### 2.3. Data Extraction and Curation

Data were extracted from the selected articles into a Microsoft Excel sheet. All articles were extracted based on the following points: first author name, year of publication, country, BVDV species and subgenotypes, reservoir, host name, the total number of animals sampled and BVDV-positive cases, prevalence or percentage, and diagnosis (including all antigen, antibody, molecular tests, and sequencing), risk factors, and prevention and control policy for BVDV. If multiple results were described in a single article, data were reviewed and cleaned to remove errors and avoid duplication.

### 2.4. Quality Assessment of the Eligible Studies

All eligible studies were subjected to a quality assessment, and the quality of each article was assessed using a scoring system (Supporting text 1). The assessment was based on nine parameters, including study design, study location, specified study period, reported prevalence or incidence, identified BVDV species, subgenotypes, diagnostic methods, specific host inclusion, and described risk factors (S. text 1). Each parameter was scored as “yes” or “no,” and two authors (Eaftekhar Ahmed Rana and Jasim M. Uddin) independently and in a blinded manner graded all the articles. A score of “1” was assigned for each “yes” answer, while a “no” was given a score of “0.” The mean score for each article was then calculated and graded as follows: low quality = 1–3, moderate quality = 4–6, and high quality = 7–9. Low-scoring studies were included, and their limitations were acknowledged in the interpretation. Since the current review did not include a meta-analysis or quantitative synthesis, a formal risk-of-bias assessment was not performed. However, potential limitations were addressed by considering key study characteristics, such as sample size, quality score, diagnostic methods, reported risk factors, and population representativeness when interpreting the results (S. text 1, S. Tables 1, and 2).

### 2.5. Statistical Analysis

Data on BVDV prevalence or incidence, animal species, BVDV species and subgenotypes, diagnostic techniques used, and risk factors were extracted and organized by country and year of publication using Microsoft Excel 2010 (Microsoft Corporation, Redmond, WA, USA). Descriptive statistics and a thematic approach were used to summarize the region-specific dominance of BVDV species and subgenotypes. No formal meta-analysis was conducted in the present review. To represent the global distribution of different species, a choropleth map was generated using ArcGIS Version 18 (Esri, Redlands, CA, USA). In addition, a heat map illustrating the diverse host range across different countries was constructed using GraphPad Prism Version 10.1.2 (GraphPad Software, San Diego, CA, USA).

## 3. Results and Discussion

### 3.1. General Data Characteristics and Quality Assessment

**Figure 2:**
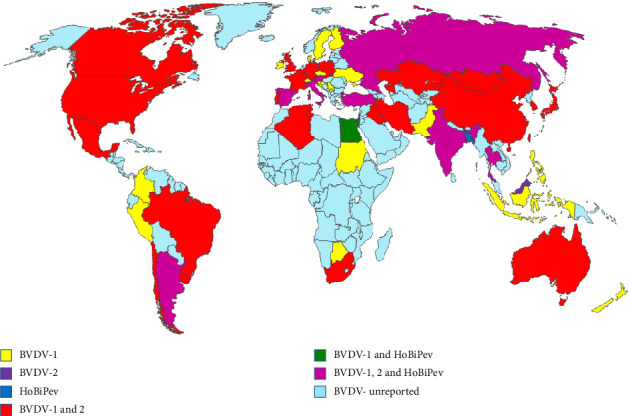
The global map illustrates the distribution of diverse species of BVDV in different countries around the world. Where BVDV-1: bovine viral diarrhea virus 1; BVDV-2: bovine viral diarrhea virus 2; HoBiPeV: HoBi-like pestivirus (previously designated as BVDV-3).

**Figure 3:**
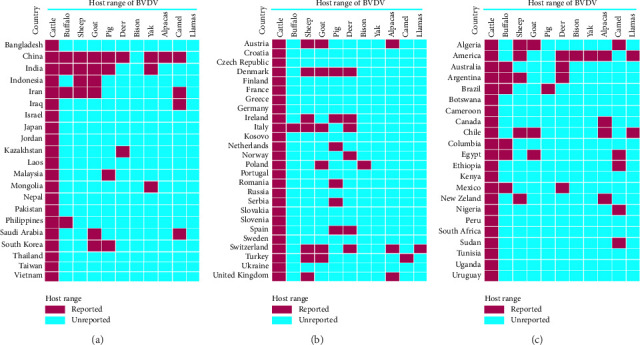
The heatmap depicts the host range of BVDV, highlighting reports of BVDV-positive cases among different domestic and wild animal species across the continents. (a) and (b) represent various Asian and European countries, respectively, while (c) illustrates countries from Africa, South America, North America, and Oceania.

A total of 1327 articles published from different countries related to BVDV infection in animals were retrieved. Of which, 248 studies were selected for this systematic review (Figure 1; Tables 1 and 2; S. Tables 1, 2, and 3). This review documented BVDV infection in 21 Asian, 26 European (EU), 11 African, 9 American (North and South), and 2 Oceanian (Australia and New Zealand) countries (Figures 2 and 3).

Based on the quality assessment, 118 studies were scored as medium quality, while 95 and 35 articles were categorized as low and high quality, respectively. In total, 248 studies were included to ensure an extensive and global representative overview of BVDV epidemiology. These studies are distinguished by their wide geographic representation, diverse hosts, and the application of multiple diagnostic and molecular techniques. Additionally, all studies contribute to a valuable understanding of BVDV genetic diversity, infection status, associated risk factors, and control strategies across diverse production systems and ecological contexts, thereby offering a unique global perspective. However, the majority of the studies are of moderate quality, which may limit the strength of study conclusions.

### 3.2. Distribution and Genetic Diversity of BVDV

Diverse species and subgenotypes of BVDV were detected in different geographical locations, as presented in Table 2 and Figure 2. BVDV is genetically heterogeneous, with genomic and antigenic variations within species and subgenotypes (Table 2). Among the 48 Asian countries, 21 countries have reported the presence of BVDV in cattle herds, but all countries have not reported at the species and subgenotype level. Twelve different subgenotypes of BVDV-1 were detected across nine countries, while two BVDV-2 subgenotypes were identified in six countries and three subgenotypes of HoBi-like pestivirus were identified in two Asian countries (Tables 2 and 3). Notably, among the Asian countries, China has described the highest genetic variability of BVDV and identified 11 BVDV-1 and two BVDV-2 subgenotypes (Table 2). China has investigated BVDV extensively compared to other Asian countries. Although all three BVDV species have been identified in Asian countries, many subgenotypes are still unexplored (Figure 2, Table 3). This gap may be attributed to resource constraints, limited molecular surveillance and characterization of circulating strains, restricted use of sequencing-based diagnostics, as well as a lack of sufficient data regarding the impact of BVDV on cattle herds. Moreover, the majority of Asian countries are economically developing, where unorganized cattle farming and marketing systems, suboptimal herd management, illegal cross-border animal trade, and poor biosecurity practices could be associated with the dissemination of high genetic diversity [33, 97]. Further, large-scale studies, including in-depth genomic analysis, are highly recommended to identify the unknown strains circulating in different cattle herds. In addition, year-round multinational BVDV surveillance and screening programs are also important to elucidate the molecular epidemiology of BVDV in Asian countries.

On the EU continent, 26 countries reported the prevalence of BVDV (S.  Table 1, Figure 2), while 23 countries reported all three species and 26 subgenotypes from different herds (Tables 1 and 2). Within the BVDV-1 species, 22 subgenotypes were explored in different countries (Table 1, Figure 2). Surprisingly, the subgenotypes BVDV-1f, 1g, 1h, 1k, 1l, 1r, 1s, 1t, 1u, and 1x were completely unique and have never been reported on other continents (Tables 2 and 3). Notably, Russia only revealed the BVDV-3a subgenotype, but within the continent, HoBi-like pestivirus is a less detected species in cattle herds (Tables 1 and 2). The current review manifests the presence of closely identical species and subgenotypes of BVDV circulating in EU countries. The presence of large free-range cattle herds might contribute to the rapid transmission and high host passages as well as mutation of viruses among the cattle population [7, 39], thus leading to the emergence of new strains or subgenotypes of BVDV. This may occur due to free animal movements and trades, as well as the exchange of animal and animals' products, such as semen [39], and less strict biosecurity programs between the countries because of open borders among EU countries. The exchange of biological products, such as fetal bovine serum, albumin, vaccines, meat, and meat products from other neighboring countries, might be associated with the introduction of emerging BVDV strains into new herds [33, 98]. However, the wider diversity of BVDV strains might pose a significant challenge to the development of effective vaccines for immunization and eradication programs in interconnected countries in Europe.

Across North and South America, only nine countries have reported evidence of 12 different subgenotypes among three BVDV species (Tables 2 and 3, Figure 2). In comparison to other continents or countries, subgenotypes of BVDV-2 (2a–2e) are predominant in Argentina, Brazil, and the United States, while BVDV-3 is a less detected species (Table 3). The distribution of different strains of BVDV is often reported as rapid genetic evolution and exchange of cattle among North and South American cattle herds [30]. However, among the 35 countries in North and South America, only a few countries have reported BVDV in cattle herds. Limited data might be associated with the limited diagnostic facility of several countries, fewer molecular surveillance studies, and a lack of awareness regarding the genetic diversity of BVDV in animal herds.

Among the 54 African countries, 11 countries have documented evidence of BVDV (Table 2 and Figure 2). Of these, six countries reported four different subgenotypes (1a, 1b, 1c, and 1j) for BVDV-1, and only Tunisia has explored BVDV-2a circulating in cattle herds. Surprisingly, none of the countries have reported the HoBi-like pestivirus species (Tables 2 and 3). The current review clearly indicates that very limited studies, lack of data, and limitations of advanced molecular studies could be responsible for the poor scenario. The reasons behind this may be the economic condition of the majority of African countries, limited resources, negligence or absence of biosecurity, uncontrolled animal movement, mixing of farm and wild animals, and lack of knowledge and awareness about the disease [90, 92]. However, further large-scale collaborative studies are essential to exploring the details of BVDV epidemiology and disease dynamics among African countries.

In the Oceanian countries, New Zealand has reported BVDV-1, while Australia has explored both BVDV-1 and BVDV-2 with a few subgenotypes in cattle herds (Tables 2 and 3). Due to their geographical position as island countries, organized cattle farming, strict biosecurity, and herd management practices might reduce the overall burden and genomic diversity of BVDV in Australia and New Zealand. Moreover, regular vaccination of cattle against BVDV, subsidized testing, and strong surveillance in dairy herds may also contribute to limiting the infection burden in herd environments [93, 95]. The greater genomic variability and widely distributed genetic diversity with a high infection burden might create serious challenges to curb and eradicate the BVD disease. To our knowledge, this is the first review to present the global distribution of circulating BVDV subgenotypes across diverse hosts. There is no doubt that accumulating information on recent circulating species and subgenotypes in different geographical locations greatly contributes to the understanding of the evolution, distribution, and molecular epidemiology of BVDV in cattle herds.

### 3.3. Host Range and Infection Scenario in Diverse Species

Although cattle are the primary host for BVDV, the virus has the capacity to infect a wide range of animal species. The BVDV has been identified in diverse domestic and wild animals around the world (S.  Table 1, Figure 3). The susceptible species include cattle, buffalo, sheep, goats, pigs, bison, captive and wild cervids, as well as Old-World and New-World camelids (NWC) (S.  Table 1, Figure 3). The susceptibility and clinical condition of BVDV vary from asymptomatic to severe disease depending on the infected host and the genetic makeup of BVDV.

#### 3.3.1. BVDV in Cattle

The seroevidence and genetic diversity of BVDV in cattle have been documented globally (S.  Table 1, Figures 2 and 3), with infection rates varying significantly. In China, prevalence ranges from 2% to 44.7% in cattle, with some studies reporting as high as 89.49% in dairy cattle and 63.27% in beef cattle [1, 15, 16, 99, 100]. Lower prevalence was evidenced in Japan and Korea, ranging between 0.2% and 5.5% [22, 101, 102]; however, some studies reported a higher prevalence in cattle herds, ranging between 26% and 58% [10, 23]. Several studies were conducted in Mongolia, Iraq, Indonesia, Vietnam, and India, where the prevalence of BVDV in cattle ranges between 11.7% and 24.6% [24, 29, 103–106]. Pakistan and Thailand reported significantly higher prevalences of 41.6% and 62.5%, respectively [14, 107]. In Bangladesh, over 51% of cattle were seropositive [97], whereas Israel (3.7%), Nepal (7.76%), and Laos (10%) showed lower rates [35, 108, 109]. Both Kazakhstan and Iraq reported a prevalence of 79% [13, 110], while Iran shows 18.6%–66.8% [111–113]. The seropositivity rates of BVDV in Jordan, Malaysia, and Saudi Arabia are reported as 31.6%, 33%, and 25.9%, respectively, in dairy herds [114–116]. Differences in prevalence may be influenced by sampling methods, detection techniques, cattle density, and biosecurity practices.

The prevalence and diversity of BVDV in cattle vary across EU countries and are summarized in S.  Table 1 and Figures 2 and 3. In Europe, BVDV seropositivity varies widely: Romania (41.4%), Spain (45.5%), France (47.9%), Poland (73.3%), Switzerland (60%–80%), and Croatia (87.1%) report high rates [38, 43, 117–120]. Lower prevalence was found in Spain, Ukraine, and Greece (∼20%) and Austria (2.9%) [52, 121, 122], whereas varied prevalence was observed in Slovenia (15.8%), Russia (29.0%), Ireland (25%), and Turkey (24.75%) [37, 123–125]. Although BVDV was detected in Italy, Germany, Poland, Peru, Slovakia, Denmark, and the United Kingdom, seroprevalence was not well documented [40, 44, 47, 54, 59, 62, 77, 126], underscoring the need for standardized nationwide surveillance programs.

In the United States, farm-level seroprevalence was reported between 12.9% and 54.7% [127, 128]. However, the prevalence of PI cattle is notably lower, typically estimated to be less than 1% of the entire cattle population in the United States [129]. Moreover, antigen detection using reverse transcriptase polymerase chain reaction (RT-PCR) tests demonstrated that BVDV-positive cattle ranged from 7% to 59.5% in Colombia and Canada [75, 130–132]. Scott et al. [133] investigated the genotype-specific prevalence in Canada using the virus neutralization test (VNT), reporting rates of 28.4% and 8.9% for BVDV-1 and BVDV-2, respectively. Both Mexico and Brazil reported more than 43% of seropositive cases of BVDV in dairy herds [134, 135]. Chile and Uruguay exhibit lower prevalence (∼1.7%–1.8%) [136, 137], though specific rates remain uncalculated in Argentina [72]. In Uruguay, BVDV prevalence reaches 55%–73% in different cattle age groups [138].

Several studies were conducted in African countries (summarized in S.  Table 1, Figures 2 and 3). Ethiopia reports an 15.4%–81% infection rate [139–141], while Nigeria (66.4%), Botswana (53.6%), Algeria (58.9%), Kenya (36.2%), Uganda (34.8%), and Cameroon (30%) also reported a higher prevalence rate in the cattle population [87, 88, 142–145]. However, Egypt and Sudan reported similar prevalence rates of 11.05% and 10.7%, respectively [84, 89]. Although only a limited number of African countries have reported BVDV, the high prevalence observed in several regions highlights the urgent need for broader surveillance, particularly in livestock-rich nations.

In Oceania, Australia exhibits a high seropositivity rate (74.5%) in the Northern Territory [146], with 0.24% of PI cattle existing in dairy farm environments in Australia [147]. Surprisingly, more than 60% of beef and dairy herds were found to be positive for BVDV infection in the southeast region of Australia [148]. Besides, 60% of dairy cattle were reported to be BVDV-seropositive in New Zealand [149]. However, studies on BVDV in this region are limited, emphasizing the need for extensive surveillance programs.

The infection rate of BVDV greatly varies globally, which might be influenced by cattle farm density, greater susceptibility of hosts, uncontrolled animal movements and trading, a lack of immunization, failure to early detection of reservoir or PI animals, neglected biosecurity, poor management practices, etc. [148]. PI animals are a well-documented reservoir of BVDV, and the presence of undetected PI animals in herds can uplift the infection rate in cattle and other animals [146]. Moreover, PI breeding bulls shed BVDV in their semen, which might be contributed to PI calves and increases the infection burden in breeding programs [111]. Environmental conditions, such as humidity and wildlife interactions, may also contribute to transmission. Additionally, sharing pasture and interacting with wildlife may increase the risk of BVDV cross-transmission, contributing to its high prevalence [16]. Contributing factors include poor management, lack of vaccination, and limited veterinary infrastructure, highlighting the need for eradication programs. Hence, the current review might contribute to understanding the overall disease scenario of BVDV in cattle herds.

#### 3.3.2. BVDV in Buffaloes

Buffaloes are a documented species infected with BVDV worldwide, although limited studies have been conducted to unveil BVDV infection (S.  Table 1, Figure 3). The prevalence of BVDV in water buffaloes in China, Iran, Colombia, and Italy was reported to be 19.40%, 20.4%, 21.7%, and 33.3%, respectively [1, 112, 150, 151]. In Laos and Australia, around 4.5% of seropositive cases were reported [109, 146], while in Brazil, 15.9%–36% of the infection rate was reported in water buffaloes [152, 153]. A study in Egypt [84] reported a 9.3% prevalence in buffaloes, whereas an earlier study reported a higher infection rate of 46.29% [154]. Although BVDV was detected in individual buffaloes in India, Mexico, and Argentina using Antigen-capture enzyme-linked immunosorbent assays (Ag-ELISA) and RT-PCR, the estimated prevalence has not been reported yet [73, 155, 156]. As a bovine species, host tropism and closely similar host cell receptors could be implicated in the susceptibility of BVDV infection in buffalo [73]. The highly variable genetic makeup of BVDV may possess altered tropism, enhancing the virus's ability to infect new species like buffalo. Moreover, close contact between cattle and buffalo due to mixed farming or grazing on the same pasture and sharing water sources may influence cross-species transmission [73, 146]. Like cattle, buffalo can become PI if exposed to BVDV in utero [1, 154] and shed the virus, acting as a source of infection. Further studies are essential to elucidate the transmission dynamics and the pathogenic role of BVDV in buffaloes.

#### 3.3.3. BVDV in Small Ruminants (Sheep and Goats)

Several serological and molecular studies have provided evidence of widespread BVDV infection in small ruminants in different countries (S.  Table 1, Figure 3). A high infection rate of BVDV was reported in sheep flocks in Argentina, the United States, and Algeria, which were 100%, 79.6%, and 68.20%, respectively [87, 157, 158]. Evidence of BVDV in sheep and goats was confirmed in India (23.4% and 16.9%), Indonesia (7.7% and 10%), and Iran (14.09% and 21.04%) [112, 159, 160]. A study in China reported 12.2% seroprevalence in sheep and goat farms [161], while New Zealand and the United States reported 6.2% and 4% BVDV infection, respectively, in larger sheep flocks [162, 163]. Moreover, a specific study on goat flocks recorded 10.2% BVDV infection in Poland [164] and 3.5% in Saudi Arabia [165]. Several countries, including Switzerland, Turkey, Austria, Ireland, and Chile, have confirmed the presence of BVDV in the sheep and goat populations, and the prevalence is widely varied based on flock size [166–171]. Notably, Italy, Denmark, the United Kingdom, South Africa, and Egypt detected BVDV infection in sheep and goats, but did not estimate the prevalence in their respective studies [44, 54, 85, 126, 172]. It has been documented that sharing pasture and mixed grazing of small ruminants with cattle are the key reasons for BVDV transmission between species [168, 169, 172]. In small ruminants, BVDV infections are often subclinical, allowing the virus to persist unnoticed [157]. However, sheep and goats are biologically susceptible to BVDV due to similarities in cellular receptors that the virus utilizes for entry [112, 150]. The significance of clinical burden in small ruminants should not be overlooked to minimize economic loss. Large-scale sero-surveillance and molecular studies are required to depict the clear landscape of BVDV infection in small ruminants.

#### 3.3.4. BVDV in Pigs

BVDV infections in pigs are sporadic, and limited serosurveys have been conducted in domestic pigs and free-ranging wild boar (S.  Table 1, Figure 3). Korea, China, and India reported 32.14%, 23.6%, and 4.86% pig farms, which were found seropositive for BVDV infection, respectively [18, 173, 174]. The clinical case of BVDV was detected in a swine herd in Malaysia [21], but the infection rate was not estimated. In Serbia, 8% of pigs carried BVDV infection [175], whereas in Spain, a pig herd was screened and found seronegative for BVDV [118]. Several studies have documented varying prevalence rates of BVDV based on antibody detection in pigs, such as 6.8% in Denmark, 3.2% in Ireland [170, 173], and 2.5% in the Netherlands [176]. The infection rate varies between 3% and 40% in Austria and Germany [177], whereas it was found to be 3.0% in Canada [178]. A very recent study in the United States reported that significant percentages (16.7%) of free-range wild pigs showed seropositivity for BVDV [67]. The prevalence of BVDV in swine herds in Ontario, USA, has been documented to range from 2% to 43% [178]. Furthermore, it was found that 64% of Brazilian swine herds tested seropositive for BVDV [179]. The prevalence of BVDV infection in pigs varies depending on geographical location, and high pig density likely contributes to higher transmission rates, mixed farming practices, and biosecurity measures in each place. Possible direct or indirect contact with ruminant species or exposure to contaminated environments might result in the transmission of BVDV in pigs [67, 179]. Therefore, highly accurate and sensitive diagnostic methods are crucial to differentiate Classical swine fever virus (CSFV) from BVDV, ensuring the correct assessment of BVDV infection status and its associated challenges in pig farming.

#### 3.3.5. BVDV in Wild Animals

Very limited studies have been conducted on BVDV infection in free-ranging wildlife populations, such as deer, bison, and yak (S.  Table 1, Figure 2). Among the Asian countries, Kazakhstan reported that 19.1% of deer were infected with BVDV [13], whereas China confirmed the clinical cases of BVDV in deer [180]. A significant percentage of white-tailed deer were found seropositive in Spain (19.5%) and Mexico (63.5%) [118, 156, 181]. Moreover, in Australia (3%), Denmark (0.63%), Switzerland (1.7%), and Norway (1.1%), very low infection rates were reported [182–186]. Italy, Ireland, and Argentina detected the BVDV in free-range deer, but did not estimate the prevalence rate [126, 187, 188]. However, several studies in the United States revealed a high percentage of deer infected with BVDV and infection rates ranging from 5.5% to 100% [189–194]. Interestingly, the BVDV was detected in skin samples from white-tailed deer in the United States, with subsequent isolation of the Cp and Ncp biotypes [194–196].

In the case of bison, a high prevalence of BVDV was reported only in America (55.3%) and Poland (29.5%) [197–199]. The presence of BVDV has been reported in Yaks with varying percentages in China (45.38%), Mongolia (20.0%), and other countries, such as India and America, without statistical estimates [1, 29, 200–202]. Surprisingly, none of the South American and African countries have reported BVDV infections in deer, bison, yaks, wildebeest, or other wild animal species. The PI animals in wildlife environments are likely to play a potential role in the establishment of wildlife reservoirs that contribute to the transmission and maintenance of BVDV infection [203]. Once the wildlife PI of BVDV is established, cross-species transmission might pose a high infection burden in wildlife environments. Notably, there is an absence of BVDV infection reports for primate, equine, canine, and feline species. Hence, an extensive study among diverse free-range wildlife populations is highly essential to exploring the epidemiology, infection burden, and genomic insight into BVDV. However, capturing wildlife for blood or tissue samples is challenging, invasive, and stressful, and requires specialized permits and equipment, adding to logistical complexities. Furthermore, limited awareness of BVDV's impact on wildlife and ecosystems diminishes the priority of surveillance efforts. The persistence of BVDV is further challenged by risks from wildlife reservoirs, which can undermine eradication efforts by serving as sources of reinfection for domestic herds. To the best of our knowledge, this is the first review study to integrate BVDV infection across wildlife species, highlighting their infection burden and the challenges of broad surveillance.

#### 3.3.6. BVDV in NWC and Old-World Camelids

BVDV infections have been observed in both Old-World camelids and NWC, including camels, alpacas, and llamas (S.  Table 1, Figure 3), which often act as asymptomatic carriers of BVDV [204]. Iraq, Iran, China, and Saudi Arabia have documented several reports of BVDV infection in Bactrian camels, with infection rates ranging between 13.63% and 30% [112, 165, 205–208]. However, a high infection rate was observed in camels from African countries, such as Egypt (27.2%), Nigeria (31.1%), Algeria (41.4%), and Sudan (84.6%) [209–212]. In contrast, Ethiopia described a lower prevalence at 2.29%, though the majority of the countries did not investigate the infection in camels [139]. In Turkey and Romania, BVDV has been detected in camels, but its prevalence at the population level remains unreported [213, 214].

The presence of BVDV has been reported in South American domestic NWCs (S.  Table 1, Figure 3). In Southern California, 25.4% of tested alpacas exhibited seropositivity, with higher antibody titers against BVDV-1 than BVDV-2 [215]. Similarly, both BVDV-1 and BVDV-2 species have been isolated from alpacas (10.8%) and lamas (4.6%) in Chile [216]. BVDV infection was reported in alpaca in Canada (7.69%), including the first record of a PI alpaca [204], whereas in the United Kingdom and the United States, BVDV was detected in 100% tissue samples collected from the ill-thrift and stillborn alpaca [216, 217]. The Cp BVDV was isolated from a stillborn llama in the United States [218, 219]. PI in alpacas has been documented in the tissues of a late-pregnant adult emaciated juvenile llama [171]. Moreover, Danuser et al. [166] detected 4.6% seroprevalence of BVDV in both alpacas and lamas in Switzerland. In contrast, China, New Zealand, and Austria reported lower prevalence of BVDV infection in alpacas, which were 3.25%, 2.1%, and 0.2%, respectively [220–222]. These reports from different regions are evidence that BVDV infections in camelids are epidemic in nature. Camelids often share environments with cattle and small ruminants, especially in mixed farming systems, which facilitates the transmission of BVDV from infected ruminants to camelids.

### 3.4. Diagnostic Approaches of BVDV and Its Limitations

Clinical diagnosis of BVDV infection from host samples is often complex and critical due to its asymptomatic and PI nature of infection. However, multiple standard laboratory tests are being used in different countries to identify the presence of virus antigen or specific antibody in host samples (S.  Table 1). Ag-ELISA, immunohistochemistry (IHC), and electron microscopy are used for the detection of BVDV-specific antigens [223, 224]. However, a critical drawback of antigen detection methods is their inability to detect low levels of antigens, particularly in late infections of BVDV, while IHC and electron microscopy are impractical for routine on-site diagnosis at the farm level. Besides, nucleic acid hybridization, RT-PCR, quantitative RT-PCR (qRT-PCR), and reverse transcriptase-loop-mediated isothermal amplification (RT-LAMP) are currently used for molecular detection of viral antigen [223, 225]. Therefore, portable field-deployable RT-PCR and RT-LAMP platforms are highly essential for on-site early detection and continuous monitoring of BVDV, particularly for timely identification of PI animals, thereby supporting effective control and eradication efforts across diverse farming systems. Moreover, recent advanced methods clustered regularly interspaced short palindromic repeat (CRISPR)/Cas12a-based platform introduced a rapid and reliable detection of different species and subgenotypes of BVDV [226]. Future development of CRISPR-based assays holds promise for even faster, highly sensitive, and field-adaptable diagnostics, further enhancing BVDV surveillance and management. However, these methods often require specialized equipment, a high level of technical expertise, a specific primer set and laboratory facilities, which may not be readily available on farm settings. Additionally, isolation and propagation of BVDV from clinical samples using susceptible cell culture systems are considered the gold standard method for diagnosis (Table 4). It is important to note that virus isolation using cell culture is time-consuming and costly, and requires a laboratory with specific cell culture capacity for BVDV-susceptible cell lines. Antibody (Ab) detection ELISA, VNT, and serum neutralization test (SNT) are performed to diagnose BVDV-specific antibodies in infected or recovered animals [227]. Among the different methods, Ag or Ab-ELISA is more frequently used (S.  Table 1), for primary screening and surveillance of BVDV in animal herds in different countries. However, ELISA tests are constrained by their inability to differentiate acute, chronic, and past infections, as well as by potential cross-reactivity with other pestiviruses when diagnosing BVDV in different species. While ELISA remains practical for large-scale herd screening due to its low cost and ease of use, these diagnostic limitations can introduce variability in prevalence estimates across regions. Therefore, to obtain more reliable and accurate epidemiological data, there is a critical need for confirmatory molecular approaches. Notably, identification of BVDV-specific genes (either the 5′ UTR, Npro, or E2 gene) by PCR is still an accurate, highly sensitive, and confirmatory method for diagnosis [225, 227]. Meanwhile, species-specific VNT and genome sequencing are currently used for specific genotyping of BVDV. However, VNT and genome sequencing for BVDV genotyping are limited due to high costs, complexity, and the need for specialized laboratory infrastructure and bioinformatics analysis, making them impractical for routine on-site farm diagnostics. Nevertheless, the diagnostic efficacy of various laboratory tests varies significantly in terms of sensitivity, specificity, accuracy, duration of test, cost, and their capability to detect the presence of BVDV or its antibodies across diverse clinical samples (Table 4). Therefore, diagnostic efforts should focus on early detection and preventing BVDV infection at the individual animal level by using cost-effective, quick, and reliable methods.

### 3.5. Risk Factors Associated With BVDV Infection

Multiple risk factors are associated with BVDV transmission, infection, and persistence in cattle herds across the globe. The risk factors reported in different countries across the world were screened and broadly categorized into host-related, herd management, biosecurity, animal breeding, human, and environmental factors (S.  Table 2).

#### 3.5.1. Host-Related Factors

Animal demography factors, such as breed, weaned calve, age and parity, lactation period, and pregnancy status, have been reported to be associated with the susceptibility to BVDV infection in different countries (S.  Table 2). Natural susceptibility to BVDV in a cattle breed may vary according to genetic traits, herd environments, and biosecurity practices [228]. Calves represent a critical risk group for BVDV as in utero infection during early gestation may result in PI animals that act as lifelong virus shedders [5], while their frequent close contact with other young stock or PI animals further amplifies their risk of exposure and transmission. Moreover, weaning is a stressful period for calves due to sudden nutritional transitions, making them more susceptible to infections, such as BVD and respiratory infections [229]. Due to the high production burden in terms of lactation and pregnancy, the animals are more vulnerable to immunosuppression and infections. Moreover, the history of repeat breeding, abortion, contact with aborted fluid, calf with a congenital defect, and previous respiratory disease are reported as risk factors for BVDV in different studies [141, 229–232].

#### 3.5.2. Herd Management Factors Contributing to BVDV

Larger herds, high animal density, and intensive farming systems are reported as risk factors for BVDV infection in dairy production systems in different countries (S.  Table 2). Mishra et al. [31] documented that mixed farming practices, where different animal species share the same housing, pasture, and management, can facilitate the cross-transmission of BVDV. In India, Kumar et al. [106] revealed that the housing system for large-scale farming is also a contributing factor to BVDV. The management of large-scale herds is often complex, as overcrowding and close contact between different categories of PI animals may facilitate the faster transmission of BVDV.

#### 3.5.3. Biosecurity Strategies Influencing BVDV

The breach of farm biosecurity practices is potentially associated with the risk of transmission of infectious diseases, including BVDV. Several studies have identified farm animals when grazing on common pasture, cattle grazing with small ruminants or communal grazing, pasture land shared by other farms, grazing on alpine pastures, and contact with wildlife animals as risk factors in different countries (S.  Table 2). Sharing common pasture land by different ruminant species could contaminate the grazing surfaces by PI animals. During the grazing of naïve animals, it may increase the likelihood of direct exposure to BVDV and become infected [232]. Moreover, animal movement factors in terms of purchase and introduction of new animals to herds, movement of cattle for marketing, movement of animals between herds, exchange of animals, and transfer of pregnant heifers between farms have been documented as risks for BVDV [98, 114, 228, 233–235]. Without quarantine and laboratory screening, the introduction of asymptomatic PI animals in farm environments may initiate the primary infection and contaminate the BVDV-free herd. Moreover, the sharing of tools and equipment that are used on PI animals and contact with farm workers are also documented as risk factors in dairy herds in several EU countries [123, 236]. Farm workers who move between different farms on the same day without maintaining biosecurity hygiene can carry BVDV on their clothing, footwear, and hands after handling PI animals or contaminated equipment [123]. Workers who have frequent and close contact with animals, performing activities, such as assistance during calving, handling sick animals, managing farm waste, feeding, and milking, can easily transmit the virus if proper biosecurity measures are not practiced.

#### 3.5.4. Animal Breeding Policies Affecting BVDV

Artificial insemination using frozen semen and natural breeding have been identified as risk factors for BVDV transmission in cattle industries (S.  Table 2). If breeding bulls carried BVDV infection, the virus can be shed through their semen during natural breeding or semen collected for artificial insemination [237]. BVDV can survive the freezing and thawing processes [237, 238], making frozen semen a potential vehicle for virus transmission. Asymptomatic breeding bulls can introduce the virus directly into the reproductive tract of cows, leading to infection. Therefore, rigorous prebreeding testing of bulls and certification of semen for BVDV-free status are essential to safeguard herd biosecurity. Furthermore, as semen trade constitutes a major route for the global exchange of bovine genetics and breed improvement, the enforcement of strict sanitary standards and official accreditation is essential to mitigate the risk of transboundary BVDV transmission.

#### 3.5.5. Human Factors Facilitating BVDV

Veterinarian and artificial inseminator, reuse of needles, rectal palpation, and farm technicians have been identified as risk objects for BVDV transmission in various countries (S.  Table 2). Veterinarians or AI practitioners use various tools for diagnosis, treatment, and breeding purposes, particularly thermometers, syringe needles, hand gloves, an AI gun, etc. However, if these contaminated tools, particularly AI guns, are reused without proper sterilization between cyclic animals, they can become a vehicle for BVDV transmission [237]. Very often, veterinarian personnel serve multiple farms in a single day and inadvertently carry the virus as a mechanical vector, which can expose numerous herds to BVDV.

#### 3.5.6. Environmental Constraints for BVDV

Geographical location and origin of animals, season, altitude, untreated manure, and close location of the manure pit to the farm are also reported as risk factors for BVDV (S.  Table 2). Cattle farms located in areas with a high incidence of BVDV are at greater risk of infection due to higher exposure rates [239]. Environmental factors, such as seasonal change and altitude, influence the temperature and humidity of particular regions, which enhance virus survival and transmission rates [240]. BVDV-infected animals shed the virus through discharge and feces [237], which can contaminate the farm environment, including pastures and water sources, leading to indirect transmission of BVDV.

Therefore, precise identification and assessment of potential risk factors associated with BVDV is highly crucial for understanding disease epidemiology, transmission dynamics, and developing biosecurity management. Moreover, this epidemiology knowledge helps in policy development for the successful control and eradication of BVDV burden in cattle herds.

### 3.6. Critical Strategies for Preventing and Controlling BVDV

The prevention and control of BVDV are challenging due to its ability to cause PIs, which serve as continuous active sources of transmission. While the epidemiology and risk factors associated with BVDV infection in dairy herds are largely uniform, the strategies for prevention and control may vary across countries due to differences in management practices, animal source, environmental conditions, and vaccination protocols. However, a few selected Asian countries, namely Indonesia, India, Japan, and China, adopted closely similar prevention and control strategies to curb BVDV (S.  Table 3). Moreover, a few countries are conducting year-round surveillance using bulk tank milk testing through Ag-ELISA and RT-PCR [224, 228, 241–243] (S.  Table 3). Although many countries have identified cases of BVD, they have yet to develop their national prevention and control strategies. This may be due to economic constraints, lack of veterinary facilities, government initiatives, expertise, and lack of knowledge about the disease. It remains unclear whether they have adopted the practices of other countries' preventive strategies or control programs.

Most of the EU, American, and Oceanian countries have implemented almost similar prevention and control strategies aiming at stamping out the disease. Notably, Norway, Denmark, Finland, Austria, and Switzerland have already eradicated the BVD successfully by implementing national guidelines [236, 244]. Conducting year-round robust surveillance programs utilizing sensitive diagnostic tests, culling of PI animals, and implementing strict biosecurity policies, including restricted animal movements, are major components of the current eradication program [4, 245–247]. Besides, several EU and Oceanian countries vaccinate cattle herds year-round (S.  Table 3). Although live-attenuated and killed vaccines are found to be effective, these vaccines still fail to prevent the establishment of PI animals [248]. Development of a more effective and advanced multivalent vaccine using circulating strain is highly recommended to produce lifelong immunity against BVDV [249].

It is recommended that early detection and removal of PI calves immediately after birth, combined with strict isolation and quarantine of newly introduced animals using highly sensitive molecular assays, be prioritized as the first line of defense to prevent herd-level transmission [249]. Furthermore, a strategic mass vaccination program at the herd level in BVDV endemic region would be essential until eradication is achieved. Notably, animal producers, traders, veterinarians, and policymakers should play coordinated roles to impetus the BVDV prevention and control program. Moreover, mandatory systematic strategies including regular surveillance, standard biosecurity practice, and awareness campaigns could be significant approaches for the prevention and eradication of BVDV.

## 4. Conclusions

This review provides a comprehensive overview of the genetic variability and the emergence of three different BVDV species and 25 subgenotypes among diverse animal species, contributing to address global knowledge gaps. In addition to cattle, various domestic ruminants and nonbovine species (such as pigs and camelids), as well as wildlife species (including bison, yak, deer, and wild boar), have been documented as being infected with BVDV in different countries. The evidence underscores that BVDV does not maintain strict host specificity, and cross-species spillover is well documented. Diagnostic discrepancies could be reduced with a global consortium because a proper diagnostic approach is pivotal for monitoring, surveillance, and successful control strategies for BVDV infection. Key measures for controlling and eradicating BVDV include culling of PI animals and avoiding mixed farming practices. The economic impact of this pathogen is unprecedented, underscoring the urgent need for proper action and coordinated multinational biosecurity measures to mitigate its effects.

### 4.1. Limitations of the Review Study

This review has several limitations. Firstly, meta-analysis was not performed due to high variability in study designs, which limited direct quantitative analysis as the study primarily focused on qualitative data, including genetic diversity, host ranges, diagnostic methods, risk factors, and control measures, which lack the quantitative consistency required for meta-analysis. Secondly, the quality scores of the eligible studies varied significantly, which may have limited the ability to equally represent BVDV data across different regions of the world, potentially affecting the accuracy of the summaries and conclusions. This limitation was necessary to restrict the breadth of the analysis but may have led to an incomplete representation of the available literature.

### 4.2. Future Recommendation

The current review highlights a significant gap in the understanding of the molecular epidemiology and genetic diversity of BVDV in cattle and other animal species, including wildlife species. As BVDV is endemic, conducting extensive genomic surveillance is highly time-demanding in regions where the virus and its circulating genetic types remain largely unexplored in both domestic and wildlife populations. Moreover, BVDV is well documented in various wildlife species, and future control programs should integrate wildlife monitoring to better understand potential spillover or reintroduction risks when developing prevention strategies. Furthermore, we recommend evaluating the cross-protective efficacy of BVDV vaccines against the diverse subgenotypes. If protection is found to be limited, the development of next-generation multivalent vaccines will be essential to ensure effective immunization, prevent cross-species transmission, and facilitate herd-level clearance of natural infection.

## Figures and Tables

**Table 1:** Keywords or terms used for the systematic search of scientific studies related to BVDV in the current systematic review.

Targeted topics for systematic search	Search terms or keywords
Virus- and disease-related search terms	(Bovine viral diarrhea virus OR, BVDV OR BVDV-1 OR BVDV-2 OR BVDV-3 OR HoBi like pestivirus OR HoBiPeV OR bovine diarrhea OR BVD OR mucosal disease, OR MD OR bovine pestivirus) AND
BVDV genetic diversity or molecular diversity	(Genetic diversity OR genetic heterogeneity OR molecular diversity OR genomic types OR genotype OR sub-genomic types OR subgenotypes OR genomic variability OR genomic characterization OR genomic surveillance OR phylogenetic analysis OR phylogeny) AND
Host infected by BVDV or susceptible host	(Host diversity OR host range OR BVDV susceptible host OR Cattle OR Buffalo OR Sheep OR Goats OR Pigs OR Swine OR Deer OR Bison OR Yak OR Camelids OR Camel OR Alpacas OR Lamas OR Wildlife Animals and other species) AND
Prevalence and epidemiology of BVDV infections	(prevalence OR seroprevalence OR occurrence OR incidence OR frequency OR infection rate OR persistent infections OR PI claves OR PI animals) AND
BVDV diagnostic tests and methods	(diagnostic approach”, OR “diagnostic test” OR Diagnostic techniques OR Detection methods OR Serological assays OR Molecular diagnostics “laboratory test” OR advanced methods) AND
Risk factors associated with BVDV	(risk factors OR risk determinants OR risk assessment OR herd-level risk factors OR animal demographic risk factors OR management-related risk factors OR biosecurity-related risk factors OR environmental risk factors) AND
Prevention and control strategy related to BVDV	(prevention and control strategy OR prevention and control policy OR prevention and control guidelines OR eradication OR and eradication programs OR mitigation OR intervention OR monitoring and surveillance OR BVDV vaccination)

**Table 2:** Distribution of different species and subgenotypes of BVDV in different countries across the continents.

Continent	Country	Species/subgenotypes of BVDV			References
		BVDV-1	BVDV-2	HoBi-like pestivirus (BVDV-3)	
Asia	Indonesia	BVDV-1a	N/A	N/A	[11, 12]
	Kazakhstan	∗DSNS	DSNS	N/A	[13]
	Pakistan	BVDV-1a	N/A	N/A	[14]
	China	BVDV-1a,1b,1c,1 d,1m, 1o, 1p, 1q,1v, 1w, Chinese BVDV ZM-95 strain	BVDV-2a, 2b	N/A	[15–20]
	Malaysia	—	DSNS	N/A	[21]
	South Korea	BVDV-1a,1b,1c	BVDV-2a	N/A	[22, 23]
	Iraq	DSNS	DSNS	N/A	[24]
	Bangladesh	N/A	N/A	BVDV-3b	[25]
	Iran	BVDV-1a,1b	BVDV-2a	N/A	[26]
	Japan	BVDV-1a,1b,1c, 1n,1o	BVDV-2a	N/A	[27, 28]
	Mongolia	BVDV-1a	BVDV-2a	N/A	[29]
	India	BVDV-1b	BVDV-2a,2b	BVDV-3c, 3d	[30–32]
	Thailand	DSNS	DSNS	DSNS	[30, 33]
	Taiwan	BVDV-1a,1b	BVDV-2a	N/A	[34]
	Israel	DSNS	DSNS	N/A	[35]
	Philippines	BVDV-1b	N/A	N/A	[36]

Europe	France	BVDV-1b,1d,1e,1l, 1r,1s,1x	DSNS	N/A	[7]
	Russia	BVDV-1a,1b,1c,1d,1f,1g,1, 1j,1k,1p,1r	BVDV-2a,2b,2c	BVDV-3a	[37]
	Croatia	BVDV-1b,1d,1f	N/A	N/A	[38]
	Poland	BVDV-1b, 1d, 1e, 1f, 1g, 1r, 1s	DSNS	N/A	[39, 40]
	Turkey	BVDV-1a,1b,1c, 1d, 1f,1i	BVDV-2a	DSNS	[41, 42]
	Switzerland	BVDV-1b,1e, 1g, 1h,1k	N/A	N/A	[43]
	Italy	BVDV-1a,1b,1c, 1d,1e,1f, 1g,1h,1j, 1k,1l,1m, 1n,1o,1p, 1q,1r,1s,1t, 1u	BVDV-2c	DSNS	[30, 44, 45]
	Ireland	BVDV-1a,1b,1d,1e	N/A	N/A	[46]
	Germany	BVDV-1a, 1b, 1d, 1e, 1f, 1h, 1g, 1k	BVDV-2a, 2c	N/A	[47, 48]
	Spain	BVDV-1a,1b,1d,1e, 1f, 1g, 1h,1k,1l	BVDV-2a	DSNS	[49, 50]
	Kosovo	BVDV-1b	N/A	N/A	[51]
	Ukraine	BVDV-1b, 1f	N/A	N/A	[52]
	United Kingdom	BVDV-1a,1b,1c,1d,1e,1f,1g,1h,1i,1j,1k,1l,1m	BVDV-2a	N/A	[53, 54]
	Austria	BVDV-1a,1b,1d, 1e,1f,1g, 1h,1k	DSNS	DSNS	[55, 56]
	Czech Republic	BVDV-1b,1d, 1e,1f	N/A	N/A	[57]
	Portugal	BVDV-1a,1b,1d, 1e	DSNS	N/A	[58]
	Denmark	BVDV-1a, 1b, 1d, 1e, 1f, 1g, 1h	N/A	N/A	[59]
	Sweden	BVDV-1a, 1b, 1d	N/A	N/A	[60]
	Finland	BVDV-1d, 1f, 1j	N/A	N/A	[61]
	Slovakia	BVDV-1a, 1b, 1d,1e, 1f,1k	BVDV- 2b	N/A	[61–63]
	Serbia	BVDV-1b,1d,1f	N/A	N/A	[64]
	Slovenia	BVDV-1b,1d,1f,1g	N/A	N/A	[65]
	Belgium	BVDV-1a, 1b	DSNS	DSNS	[66]

North and South America	United States of America (USA)	BVDV-1a, 1b	BVDV-2a, 2b, 2e	N/A	[67–70]
	Mexico	BVDV-1a, 1b	BVDV-2a	N/A	[71]
	Argentina	BVDV-1a, 1b, 1e, 1i	BVDV-2a, 2b,2c, 2d	DSNS	[61, 72–74]
	Colombia	BVDV-1a	N/A	N/A	[75]
	Chile	BVDV-1a,1b,1c,1j	BVDV-2a	N/A	[76, 77]
	Canada	BVDV-1a, 1b	BVDV2a	N/A	[78]
	Brazil	BVDV-1a,1b,1d,1e, 1i	BVDV-2b,2c	N/A	[61, 79–81]
	Uruguay	BVDV-1a, 1i	BVDV-2b	N/A	[82]
	Peru	BVDV- 1b	N/A	N/A	[77]

Africa	Egypt	BVDV-1a,1b, 1j	N/A	DSNS	[83–85]
	Algeria	BVDV-1a	DSNS	N/A	[86, 87]
	Botswana	BVDV-1a	N/A	N/A	[88]
	Sudan	DSNS	N/A	N/A	[89]
	South Africa	BVDV-1a, 1b,1c	DSNS	N/A	[90, 91]
	Tunisia	BVDV-1b	BVDV-2a	N/A	[92]

Oceania	New Zealand	BVDV-1a, 1c	N/A	N/A	[93, 94]
	Australia	BVDV-1a,1b,1c	BVDV-2a	N/A	[95, 96]

*Note:* HoBi-like pestivirus (previously known as BVDV-3), DSNS = detected species, nonspecific subgenotypes, N/A = means no data available.

**Table 3:** Summary of genetic diversity (species and subgenotypes) of BVDV circulating in different continents of the world.

Continent (number of countries)	^∗^BVDV species	^∗^Circulating subgenotypes of BVDV
Asian (15)	BVDV-1	1a, 1b, 1c, 1d, 1m, 1n, 1o, 1p, 1q, 1v, 1w, Chinese ZM-95
	BVDV-2	2a, 2b
	HoBiPeV (BVDV-3)	3b, 3c, 3d

European (23)	BVDV-1	1a, 1b,1 c, 1d, 1e, 1f, 1g, 1h, 1i, 1j,1 k, 1l, 1m, 1n, 1o, 1p, 1q, 1r, 1s, 1t, 1u, 1x
	BVDV-2	2a, 2b, 2c
	HoBiPeV (BVDV-3)	3a

North and South American (9)	BVDV-1	1a, 1b, 1c, 1d, 1e, 1i, 1j
	BVDV-2	2a, 2b, 2c, 2d, 2e
	HoBiPeV (BVDV-3)	Unreported

African (6)	BVDV-1	1a, 1b, 1c, 1j
	BVDV-2	2a
	HoBiPeV (BVDV-3)	Unreported

Oceania (2)	BVDV-1	1a, 1b, 1c
	BVDV-2	2a
	HoBiPeV (BVDV-3)	Unreported

*Note:* HoBiPeV = HoBi-like pestivirus.

^∗^This has been summarized from Table 1; therefore, references have not been mentioned again in order to avoid repetition.

**Table 4:** Different diagnostic tests used to detect BVD in various clinical samples.

Target components	Clinical samples	Test name (currently used)	Test sensitivity and specificity (%)	Time required for diagnosis	Ability to detect PI animals
Cell culture and isolation of BVDV	Whole blood, serum, fecal materials (from diarrheic animals), aborted materials, uterine fluid, skin samples (ear notch), milk samples (milk leukocytes), nasal, vaginal, and rectal swab	Culture and propagation of BVDV (commonly used cell lines: Madin–Darby bovine kidney [MDBK], bovine turbinate [BT], primary bovine fetal lung cells, fetal bovine kidney [FBK], and bovine testicular cells)	Gold standard	2–14 days	Most reliable

^∗^BVDV antigen	Same as the above samples	Ag-ELISA	67–100	2–6 h	Possible
		IHC	98–100	2–4 days	Possible
		IFA	App. 100	1–2 days	Possible

BVDV-specific nucleic acid (RNA)	Same as the above (extraction of viral RNA; and targeting specific genes, such as 5′-UTR, E2, and N^pro^)	RT-PCR	App. 100	12–24 h	Reliable
		RT-qPCR	App. 100	6–12 h	Reliable
		RT-LAMP	98–100	> 1 h	Reliable
		SS and WGS	100	2–5 days	Specific
		CRISPR-Cas12a	65–100	> 1 h	Reliable

BVDV-specific antibody	Whole blood, serum, milk, and colostrum	Ab-ELISA	98–99	4–6 h	^∗^NS
		SNT or VNT	98–100	3–5 days	NS
		DbEI	98–100	1–2 days	NS
		AGID	98–100	1–2 days	NS
		MBI	99.4–98.3	2 days	NS

*Note:* ^∗^BVDV antigen (SP: structural protein and NSP: nonstructural protein), e.g., NS3 (p80) and Erns(E0); ^∗^NS: not specific (need to further antigen detection test); App: approximate; hrs: hours. Ab-ELISA, antibody-capture enzyme-linked immunosorbent assay; Ag-ELISA, antigen-capture enzyme-linked immunosorbent assay; DbEI, dot-blot enzyme immunoassay; AGID, agarose gel immunodiffusion; IFA, immunofluorescence assay; WGS, whole genome sequencing or RNA deep sequencing.

Abbreviations: IHC, immunohistochemistry; MBI, microsphere-based immunoassay; RT-LAMP, reverse transcriptase-loop-mediated isothermal amplification; RT-PCR, reverse transcription polymerase chain reaction; RT-qPCR, quantitative reverse transcription polymerase chain reaction; SNT, serum neutralization test; SS, Sanger sequencing; UTR, untranslated region; VNT, virus neutralization test.

## Data Availability

The data that support the findings of this study are available in the supporting information of this article.

## Supporting Information

Supporting Information 1 Supporting text 1: Checklist used for quality assessment and scoring of eligible articles.

Supporting Information 2 Supporting Table 1: Diverse host range, infection burden of BVDV, and diagnostic methods used in different countries of the world.

Supporting Information 3 Supporting Table 2: Different risk factors associated with BVDV infection in cattle herds documented in various countries.

Supporting Information 4 Supporting Table 3: Prevention and control strategies for BVDV implemented by different countries across the globe.
