# Supplementary material for: Molecular Epidemiology and Control Strategies for BVDV: A Global Systematic Review From 2000 to 2025
Source: Vet Med Int. 2025 Nov 12;2025:6732453. doi: 10.1155/vmi/6732453 (PMC12629698; doi:10.1155/vmi/6732453)
Supplement: Supporting Information 1 — Supporting text 1: Checklist used for quality assessment and scoring of eligible articles. [file 6732453.f1.docx]

**Supplementary text 1: Checklist used for quality assessment and scoring of eligible articles.**

The following parameters were evaluated and given a score based on a simple scale system (1 for ''yes'', 0 for ''no'') [**Ahaduzzaman**, 2020; and Ran et al., 2019].

1. Was the study design clearly stated?
2. Was the sampling area clearly described with reference to the country?
3. Was the period of the study stated?
4. Was the study reported prevalence or incidence of BVDV?
5. Was the study identified or confirmed BVDV species (BVDV-1, BVDV-2, BVDV-3)?
6. Was the study identified or confirmed sub-genotypes of BVDV species?
7. Was the sample diagnostic method clearly described?
8. Was the study mentioned specific host species used for BVDV study?
9. Was the sample described risk factors for BVDV?

**Reference**

1. **Ahaduzzaman, M.** Peste des Petits Ruminants (PPR) in Africa and Asia: A Systematic Review and Meta-Analysis of the Prevalence in Sheep and Goats between 1969 and 2018. Vet. Med. Sci. **2020**, 6(4), 813–833.
2. Ran, X.; Chen, X.; Ma, L.; Wen, X.; Zhai, J.; Wang, M.; Tong, X.; Hou, G.; Ni, H. A Systematic Review and Meta-Analysis of the Epidemiology of Bovine Viral Diarrhea Virus (BVDV) Infection in Dairy Cattle in China. Acta Trop. 2019, 190, 296–303.
