# Supplementary material for: Molecular Epidemiology and Control Strategies for BVDV: A Global Systematic Review From 2000 to 2025
Source: Vet Med Int. 2025 Nov 12;2025:6732453. doi: 10.1155/vmi/6732453 (PMC12629698; doi:10.1155/vmi/6732453)
Supplement: Supporting Information 2 — Supporting Table 1: Diverse host range, infection burden of BVDV, and diagnostic methods used in different countries of the world. [file 6732453.f2.docx]

**Supplementary Table 1: Diverse host range, infection burden of BVDV and diagnostic methods used in different countries of the world.**

| **Study reference** | **Country** | **Detection methods** | **Host identified positive for BVDV**  **Total number of animals tested (positive animals)** | | | | | | | | | **Prevalence** |
| --- | --- | --- | --- | --- | --- | --- | --- | --- | --- | --- | --- | --- |
|  |  |  | **Cattle** | **Buffalo** | **Sheep** | **Goat** | **Pig** | **Deer** | **Bison** | **Camelids** | **Yak** |  |
| **Cattle** |  |  |  |  |  |  |  |  |  |  |  |  |
| Birhanu et al., 2024 | Ethiopia | Ab-ELISA, Ag-ELISA | 337 (52) | **-** | N/A | N/A | N/A | N/A | N/A | N/A | N/A | Cattle:15.4% |
| Zhigailov et al., 2023 | Kazakhstan | Ab-ELISA, RT-PCR | 2477  (1965) | N/A | N/A | N/A | N/A | 21  (4) | N/A | N/A | N/A | Cattle: 79.3%,  Deer: 19.1% |
| Hugues et al., 2023 | Chile | Ag-ELISA, RT-PCR , Sequencing | 1331  (24) | N/A | N/A | N/A | N/A | N/A | N/A | N/A | N/A | Cattle: 1.8% |
| Karimi et al., 2022 | Iran | Ab-ELISA | 800 (535) | N/A | N/A | N/A | N/A | N/A | N/A | N/A | N/A | Cattle: 66.8% |
| Kučer et al., 2022 | Croatia | VNT, Ab-ELISA | 233  (VNT: 194;  Ab-ELISA: 203) | N/A | N/A | N/A | N/A | N/A | N/A | N/A | N/A | Cattle (VNT): 83.26%  Cattle (Ab-ELISA): 87.12% |
| Ahmad et al., 2022 | Pakistan | Ab-ELISA | 600 (250) | N/A | N/A | N/A | N/A | N/A | N/A | N/A | N/A | Cattle: 41.6% |
| Antos et al., 2021 | Poland | Ag-ELISA, RT-PCR | 45  (33) | N/A | N/A | N/A | N/A | N/A | N/A | N/A | N/A | Cattle:73.3% |
| Spetter et al., 2021 | Argentina | RT-PCR, Sequencing | Detected | N/A | N/A | N/A | N/A | N/A | N/A | N/A | N/A | Not estimated |
| Tesfaye et al., 2021 | Ethiopia | Ab-ELISA, Ag- ELISA | 219 (177) | N/A | N/A | N/A | N/A | N/A | N/A | 348 (8) | N/A | Cattle: 80.82%  Camel: 2.29% |
| VanLeeuwen et al., 2021 | Kenya | Ab-ELISA, Ag-ELISA | 467 (169) 36.2% | N/A | N/A | N/A | N/A | N/A | N/A | N/A | N/A | Cattle:36.2% |
| Toplak et al., 2021 | Slovenia | Ab-ELISA, RT-qPCR | 4756 (762) | N/A | N/A | N/A | N/A | N/A | N/A | N/A | N/A | Cattle: 15.8% |
| Caffarena et al., 2021 | Uruguay | Ag- ELISA, RT-PCR | Diarrheic calves: 231 (4) and  Non-diarrheic calves: 249 (2) | N/A | N/A | N/A | N/A | N/A | N/A | N/A | N/A | Diarrheic calves: 1.7%;  Non-diarrheic calves: 0.8% |
| Guo et al., 2021 | China | RT-PCR | 302  (135) | N/A | N/A | N/A | N/A | N/A | N/A | N/A | N/A | Cattle: 44.7% |
| Chang et al., 2021 | China | IFA, RT-PCR, TEM ,VI | 1234  (89) | N/A | N/A | N/A | N/A | N/A | N/A | N/A | N/A | Cattle: 7% |
| Deng et al., 2020 | China | Ag-ELISA, Ab-ELISA, RT-PCR , Sequencing | 901  (21) | N/A | N/A | N/A | N/A | N/A | N/A | N/A | N/A | Cattle: 2% |
| Hou et al., 2020 | China | RT-PCR | 169  (29) | N/A | N/A | N/A | N/A | N/A | N/A | N/A | N/A | Cattle:17.2% |
| McMorrow et al., 2020 | Australia | Ab-ELISA | Beef cattle:61%, Dairy herd: 53%, | N/A | N/A | N/A | N/A | N/A | N/A | N/A | N/A | Beef cattle:61%, Dairy herd: 53%, |
| Rypuła et al., 2020 | Poland | Ab-ELISA | Cattle herd: 354 (118) | N/A | N/A | N/A | N/A | N/A | N/A | N/A | N/A | Cattle herd: 33.3% |
| Guidoum et al., 2020 | Algeria | Ab-ELISA, RT-PCR | 234  (138) | N/A | N/A | N/A | N/A | N/A | N/A | N/A | N/A | Cattle: 58.9% |
| Akagami et al., 2020 | Japan | Ab-ELISA | 9016  (2378) | N/A | N/A | N/A | N/A | N/A | N/A | N/A | N/A | Cattle: 26% |
| Nugroho et al., 2020 | Indonesia | Ag and Ab-ELISA | 77( 9) | N/A | N/A | N/A | N/A | N/A | N/A | N/A | N/A | Cattle:11.7% |
| Noaman and Nabinejad, 2020 | Iran | Ab-ELISA | 216  (114) | N/A | N/A | N/A | N/A | N/A | N/A | N/A | N/A | Cattle: 52.7% |
| Berg et al., 2019 | Botswana | Ab-ELISA, Sequencing | 364  (195) | N/A | N/A | 100  (0) | N/A | N/A | N/A | N/A | N/A | Cattle: 53.6%;  Goat: 0% |
| Kim et al., 2019 | Korea | Ag-ELISA, IHC | 3050  (21) | N/A | N/A | N/A | N/A | N/A | N/A | N/A | N/A | Cattle:0.6% |
| Agah et al., 2019 | Japan | Ag-ELISA | 1075  (2) | N/A | N/A | N/A | N/A | N/A | N/A | N/A | N/A | Cattle: 0.2% |
| Thapa et al., 2019 | Nepal | Ab-ELISA | 92 (7) | N/A | N/A | N/A | N/A | N/A | N/A | N/A | N/A | Cattle: 7.76% |
| Tadesse et al., 2019 | Ethiopia | Ab-ELISA | 420  (217) | N/A | N/A | N/A | N/A | N/A | N/A | N/A | N/A | Cattle: 51.6% |
| Ricci et al., 2019 | Italy | Ag-ELISA, RT-PCR, Sequencing | Detected | N/A | Detected | N/A | N/A | Detected | N/A | N/A | N/A | Not estimated |
| Ryu and Choi, 2019 | Korea | RT-PCR | 635  (35) | N/A | N/A | N/A | N/A | N/A | N/A | N/A | N/A | Cattle: 5.5% |
| Quintero et al., 2019 | Columbia | RT-PCR, Sequencing | 7% | N/A | N/A | N/A | N/A | N/A | N/A | N/A | N/A | Cattle: 7% |
| Bulut et al., 2018 | Turkey | RT-PCR | 396  (98) | N/A | N/A | N/A | N/A | N/A | N/A | N/A | N/A | Cattle: 24.75% |
| Kumar et al., 2018 | India | Ab-ELISA | 500  (66) | N/A | N/A | N/A | N/A | N/A | N/A | N/A | N/A | Cattle: 13.2% |
| Han et al., 2018 | Korea | RT-PCR, Sequencing | 149  (87) | N/A | N/A | N/A | N/A | N/A | N/A | N/A | N/A | Cattle: 58% |
| Olmo et al., 2018 | Laos | Ab-ELISA | 90  (9) | 61  (3) | N/A | N/A | N/A | N/A | N/A | N/A | N/A | Cattle:10%,  Buffalo: 4.9% |
| Reichel et al., 2018 | New Zealand | Ab-ELISA, | 60% | N/A | N/A | N/A | N/A | N/A | N/A | N/A | N/A | Cattle: 60% |
| Hasan and Alsaad, 2018 | Iraq | Ag-ELISA, RT-PCR | 494 (69) | N/A | N/A | N/A | N/A | N/A | N/A | N/A | N/A | Cattle:13.96% |
| Stalder et al., 2018 | Switzerland | Ab-ELISA, RT-PCR, Sequencing | 3440 (1982) | N/A | N/A | N/A | N/A | N/A | N/A | N/A | N/A | Cattle: 57.61% |
| Viana et al., 2017 | Brazil | Ab- ELISA, VNT | 400  (157) | N/A | N/A | N/A | N/A | N/A | N/A | N/A | N/A | Cattle: 39.35% |
| Decaro et al., 2017 | Italy | Ab- ELISA, RT-PCR, Sequencing | 44 farm positive | one farm positive | 21 flocks positive | 6 flocks positive | N/A | N/A | N/A | N/A | N/A | Not estimated |
| Wolff et al., 2017 | Uganda | Ab- ELISA | 138 (48) farm positive | N/A | N/A | N/A | N/A | N/A | N/A | N/A | N/A | Cattle: 34.8% |
| Wernike et al., 2017 | Germany | Ag-ELISA, RT-PCR, Sequencing | Detected | N/A | N/A | N/A | N/A | N/A | N/A | N/A | N/A | Not estimated |
| Uddin et al., 2017 | Bangladesh | Ab-ELISA | 94  (48) | N/A | N/A | N/A | N/A | N/A | N/A | N/A | N/A | Cattle: 51% |
| Kaveh et al., 2017 | Iran | RT-PCR | 128  (26) | N/A | N/A | N/A | N/A | N/A | N/A | N/A | N/A | Cattle: 20% |
| Rodrígue-Prieto et al., 2016 | Spain | Ab-ELISA, RT-qPCR | 180  (82) | N/A | N/A | N/A | 62  (0) | 267  (52) | N/A | N/A | N/A | Cattle: 45.5%,  Deer: 19.5%  Pig:0% |
| Daves et al., 2016 | Malaysia | Ab-ELISA | 407  (135) | N/A | N/A | N/A | N/A | N/A | N/A | N/A | N/A | Cattle: 33% |
| Hay et al., 2016 | Australia | Ab- ELISA, RT-qPCR | PI Cattle:35131 (PI:85) | N/A | N/A | N/A | N/A | N/A | N/A | N/A | N/A | PI Cattle: 0.24% |
| Segura-Correa et al., 2016 | Mexico | Ab- ELISA | 385  (184) | N/A | N/A | N/A | N/A | N/A | N/A | N/A | N/A | Cattle: 47.8% |
| Evans et al., 2016 | Australia | Ab- ELISA | 184 (137) | 245 (11) | N/A | N/A | N/A | N/A | N/A | N/A | N/A | Cattle: 74.5%  Buffalo: 4.5% |
| Glotov et al., 2016 | Russia | Ab- ELISA, RT-PCR | 29.0 % | N/A | N/A | N/A | N/A | N/A | N/A | N/A | N/A | Cattle: 29.0 % |
| Ochirkhuu et al., 2016 | Mongolia | Ag-ELISA, VI, RT-PCR, IHC, Sequencing | 19 (3) | N/A | N/A | N/A | N/A | N/A | N/A | N/A | 20.0 % | Cattle:15.8 %  Yak: 20.0 % |
| Bello et al., 2016 | Nigeria | Ab- ELISA | 372 (247) | N/A | N/A | N/A | N/A | N/A | N/A | N/A | N/A | Cattle: 66.39% |
| Nilnont et al., 2016 | Thailand | Ab- ELISA | 933(584) | N/A | N/A | N/A | N/A | N/A | N/A | N/A | N/A | Cattle: 62.5 % |
| Sayers et al., 2015 | Ireland | Ab-ELISA | 2171  (543) | N/A | N/A | N/A | N/A | N/A | N/A | N/A | N/A | Cattle: 25% |
| Kulangara et al., 2015 | India | Ab-ELISA, RT-PCR | 385  (95) | N/A | N/A | N/A | N/A | N/A | N/A | N/A | N/A | Cattle: 24.6% |
| Soltan  et al., 2015 | Egypt | RT-PCR, RT-qPCR, Sequencing | 190 (21) | 108 (10) | N/A | N/A | N/A | N/A | N/A | N/A | N/A | Cattle: 11.05%  Buffalo: 9.25% |
| Saeed et al., 2015 | Sudan | Ag-ELISA, FAT, RT-PCR, Sequencing | 242 (26) | N/A | N/A | N/A | N/A | N/A | N/A | N/A | N/A | Cattle: 10.7% |
| Deng et al., 2015 | China | Ag-ELISA, Ab-ELISA, RT-PCR , Sequencing | Dairy cattle: (298/333), Beef cattle: (248/392), | 134 (26) | N/A | N/A | N/A | N/A | N/A | N/A | yaks: (236/520) | Dairy cattle: 89.49%, Beef cattle: 63.27% Water Buffalo : 19.40% ; Yak: 45.38% |
| Stegniy et al., 2014 | Ukraine | Ab-ELISA, RT-qPCR , Sequencing | 283 (57) | N/A | N/A | N/A | N/A | N/A | N/A | N/A | N/A | Cattle: 20.1% |
| Mahmoud and Allam, 2013 | Saudi Arabia | Ab-ELISA | 460  (119) | N/A | N/A | N/A | N/A | N/A | N/A | N/A | N/A | Cattle: 25.9% |
| Kuta et al., 2013 | Poland | VI, RT-PCR | Detected | N/A | N/A | N/A | N/A | N/A | N/A | N/A | N/A | Not estimates |
| Aniță et al., 2013 | Romania | Ab-ELISA | 77 (37) | N/A | N/A | N/A | N/A | N/A | N/A | N/A | N/A | Cattle: 41.44% |
| Alsaad et al., 2012 | Iraq | Ab-ELISA | 84  (66) | N/A | N/A | N/A | N/A | N/A | N/A | N/A | N/A | Cattle: 78.5% |
| Sharifzadeh et al., 2011 | Iran | RT-PCR | 172  (32) | N/A | N/A | N/A | N/A | N/A | N/A | N/A | N/A | Cattle:18.6% |
| Safarpoor Dehkordi, 2011 | Iran | RT-PCR | 620 (127) | 372 (76) | 525 (74) | 442 (93) | - | - | **-** | 214 (32) | **-** | Cattle: 20.48%  Buffalo: 20.43%  Sheep: 14.09%  Goat: 21.04%  Camel: 14.95% |
| Handel et al., 2011 | Cameroon | Ab-ELISA, Ag-ELISA, RT-PCR | 146 (44) Herd level  30% | N/A | N/A | N/A | N/A | N/A | N/A | N/A | N/A | Cattle: 30% |
| Friedgut et al., 2011 | Israel | Ab-ELISA, Ag-ELISA, RT-PCR, Sequencing | Dairy cattle: 350 (13); Calves: 1100 (66) | N/A | N/A | N/A | N/A | N/A | N/A | N/A | N/A | Cattle: 3.7%  Calves: 6% |
| Ståhl et al., 2009 | Peru | RT-PCR, Sequencing | Detected | N/A | N/A | N/A | N/A | N/A | N/A | N/A | N/A | Not estimated |
| Talafha et al., 2009 | Jordan | Ab-ELISA | 671 (207) | N/A | N/A | N/A | N/A | N/A | N/A | N/A | N/A | Cattle: 30.8% |
| Guarino et al., 2008 | Uruguay | Ab-ELISA | 6358 (2271 from heifers, 2285 from cows, and 1802 from bulls) | N/A | N/A | N/A | N/A | N/A | N/A | N/A | N/A | Heifer:55%  Cow:73%  Bull:69% |
| NOVÁČKOVÁ et al., 2008 | Slovakia | Ag ELISA, RT-PCR | Detected | N/A | N/A | N/A | N/A | N/A | N/A | N/A | N/A | Not estimated |
| Booker et al., 2008 | Canada | IHC, RT-PCR | PI Cattle: 7132 (2504);  Acute infection : 4628 (2753) | N/A | N/A | N/A | N/A | N/A | N/A | N/A | N/A | PI Cattle: 35.10%  Acute Infection: 59.5% |
| Duong et al., 2008 | Vietnam | Ag-ELISA, Ab-ELISA | 130 (23) | N/A | N/A | N/A | N/A | N/A | N/A | N/A | N/A | Cattle: 18% |
| O'Connor et al., 2007 | United States | Ag-ELISA, IHC | 12030 Calves  ( Ag-ELISA: 24,  IHC:13) | N/A | N/A | N/A | N/A | N/A | N/A | N/A | N/A | Calves: 0.2% |
| Scott et al., 2006 | Canada | VNT | 179 ( BVDV-1: 51; BVDV-2:16) | N/A | N/A | N/A | N/A | N/A | N/A | N/A | N/A | Cattle (BVDV-1): 28.4%  Cattle (BVDV-2)=8.9% |
| Billinis et al., 2005 | Greece | Ag-ELISA | 6333 (1267) | N/A | N/A | N/A | N/A | N/A | N/A | N/A | N/A | Cattle: 20% |
| Uttenth et al., 2005 | Denmark | RT-PCR, Sequencing | Detected | N/A | N/A | N/A | N/A | N/A | N/A | N/A | N/A | Not estimated |
| Wakeley et al., 2004 | UK | Ag-ELISA, RT-qPCR | Detected | N/A | Detected | N/A | N/A | N/A | N/A | N/A | N/A | Not estimated |
| Fulton et al., 2002 | United States | VNT, Ab-ELISA, Histopathology | 325 (42) | N/A | N/A | N/A | N/A | N/A | N/A | N/A | N/A | Claves : 12.9% |
| Deregt et al., 2002 | Canada | RT-PCR, VI | 450  (PCR= 47, VI= 39) | N/A | N/A | N/A | N/A | N/A | N/A | N/A | N/A | Calves: (PCR= 10.4%  VI= 8.7%) |
| Rossmanith et al., 2001 | Austria | Ag-ELISA,  RT-PCR, Sequencing | 6110 (ELISA=178, RT-PCR=213) | N/A | N/A | N/A | N/A | N/A | N/A | N/A | N/A | Cattle:  (ELISA= 2.9%  RT-PCR= 3.5%) |
| Mainar-Jaime et al., 2001 | Spain | Ab-ELISA, VNT | 529 (114) | N/A | N/A | N/A | N/A | N/A | N/A | N/A | N/A | Cattle:  21.1% |
| Rush et al., 2001 | USA | SN, RT-PCR | 446 calves (244) | N/A | N/A | N/A | N/A | N/A | N/A | N/A | N/A | Cattle: 54.7% |
| Beaudeau et al., 2001 | France | Ab-ELISA, VNT | 1189 (570) | N/A | N/A | N/A | N/A | N/A | N/A | N/A | N/A | Cattle:  47.94% |
| Haines et al., 2001 | Canada | IHC | 49 (20) | N/A | N/A | N/A | N/A | N/A | N/A | N/A | N/A | Calves: 41% |
| **Buffalo** |  |  |  |  |  |  |  |  |  |  |  |  |
| Olmo et al., 2018 | Laos | Ab-ELISA | 90  (9) | 61 (3) | N/A | N/A | N/A | N/A | N/A | N/A | N/A | Cattle:10%,  Buffalo: 4.9% |
| Decaro et al., 2017 | Italy | Ab- ELISA, RT-PCR, Sequencing | 44 farm positive | one farm positive | 21 flocks positive | 6 flocks positive | N/A | N/A | N/A | N/A | N/A | Not estimated |
| Evans et al., 2016 | Australia | Ab- ELISA | 184 (137) | 245 (11) | N/A | N/A | N/A | N/A | N/A | N/A | N/A | Cattle: 74.5%  Buffalo: 4.5% |
| Soltan  et al., 2015 | Egypt | RT-PCR, RT-qPCR, Sequencing | 190 (21) | 108 (10) | N/A | N/A | N/A | N/A | N/A | N/A | N/A | Cattle: 11.05%  Buffalo: 9.25% |
| Deng et al., 2015 | China | Ag-ELISA, Ab-ELISA, RT-PCR , Sequencing | Dairy cattle: (298/333), Beef cattle: (248/392), | 134 (26) | N/A | N/A | N/A | N/A | N/A | N/A | yaks: (236/520) | Dairy cattle: 89.49%, Beef cattle: 63.27% Water Buffalo : 19.40% ; Yak: 45.38% |
| Medina-Gudiño et al., 2022 | Mexico | RT-PCR , Sequencing | N/A | Detected | N/A | N/A | N/A | Detected | N/A | N/A | N/A | Not estimated |
| Pastrana et al., 2022 | Colombia | Ab-ELISA | N/A | 861 (187) | N/A | N/A | N/A | N/A | N/A | N/A | N/A | Buffalo: 21.7% |
| Paixão et al., 2018b | Brazil | VNT | N/A | 305  (110) | N/A | N/A | N/A | N/A | N/A | N/A | N/A | Buffalo: 36% |
| Paixão et al., 2018a | Brazil | VNT, RT-PCR | N/A | 44 (7) | N/A | N/A | N/A | N/A | N/A | N/A | N/A | Buffalo: 15.9% |
| Pecora et al., 2017 | Argentina | Ab- ELISA, VNT | N/A | Detected | N/A | N/A | N/A | N/A | N/A | N/A | N/A | Not estimated |
| Safarpoor Dehkordi, 2011 | Iran | RT-PCR | 620 (127) | 372 (76) | 525 (74) | 442 (93) | N/A | N/A | N/A | 214 (32) | N/A | Cattle: 20.48%  Buffalo: 20.43%  Sheep: 14.09%  Goat: 21.04%  Camel: 14.95% |
| Martucciello et al., 2009 | Italy | Ag-ELISA, RT-PCR | N/A | 9 (3) | N/A | N/A | N/A | N/A | N/A | N/A | N/A | Buffalo:33.4% |
| Ahmed and Zaher, 2008 | Egypt | RT-PCR | N/A | 175 (81) | N/A | N/A | N/A | N/A | N/A | N/A | N/A | Buffalo: 46.29% |
| Mishra et al., 2007 | India | Ag-ELISA, RT-PCR | N/A | Detected | N/A | N/A | N/A | N/A | N/A | N/A | N/A | Not estimated |
| **Sheep** |  |  |  |  |  |  |  |  |  |  |  |  |
| Hidayat et al., 2021 | Indonesia | Ab-ELISA; RT-PCR | N/A | N/A | 26  ( 2) | 20  (2) | N/A | N/A | N/A | N/A | N/A | Sheep: 7.7%  Goat: 10% |
| Evans et al., 2020 | New Zealand | Ab-ELISA, VNT | N/A | N/A | 270  (17) | N/A | N/A | N/A | N/A | N/A | N/A | Sheep: 6.2% |
| Ricci et al., 2019 | Italy | Ag-ELISA, RT-PCR, Sequencing | Detected | N/A | Detected | N/A | N/A | Detected | N/A | N/A | N/A | Not estimated |
| Feknous et al., 2018 | Algeria | Ag-ELISA, Ab-ELISA, RT-PCR, VNT, Sequencing | N/A | N/A | 689 (470) | N/A | N/A | N/A | N/A | N/A | N/A | Sheep: 68.20% |
| Silveira et al., 2018 | USA | VI, RT-PCR, VNT | N/A | N/A | 500  (20) | N/A | N/A | N/A | N/A | N/A | N/A | Sheep: 4% |
| Decaro et al., 2017 | Italy | Ab- ELISA, RT-PCR, Sequencing | 44 farm positive | one farm positive | 21 flocks positive | 6 flocks positive | N/A | N/A | N/A | N/A | N/A | Not estimated |
| Shi et al., 2016 | China | Ab- ELISA, RT-PCR, Sequencing | - | - | 49 (6) | 49 (6) | N/A | N/A | N/A | N/A | N/A | Sheep: 12.2%  Goat:12.2% |
| Safarpoor Dehkordi, 2011 | Iran | RT-PCR | 620 (127) | 372 (76) | 525 (74) | 442 (93) | N/A | N/A | N/A | 214 (32) | N/A | Cattle: 20.48%  Buffalo: 20.43%  Sheep: 14.09%  Goat: 21.04%  Camel: 14.95% |
| Danuser et al., 2009 | Switzerland | Ab-ELISA, VNT | N/A | N/A | 5059 (815) | 503 (128) | N/A | N/A | N/A | 109 (5) | N/A | Sheep: 16.1%  Goats: 25.4%  Alpacas and Lamas :4.6% |
| Mishra et al., 2009 | India | Ab-ELISA, Ag-ELISA, VNT | N/A | N/A | 1077 | 1026 | N/A | N/A | N/A | N/A | N/A | Sheep: 23.4%  Goat: 16.9% |
| Julia et al., 2009 | Argentina | VNT, RT-PCR | N/A | N/A | 54 (43) | **-** | N/A | N/A | N/A | N/A | N/A | Sheep:79.6% |
| Yesilbag and Gungor, 2009 | Turkey | VNT, VI | N/A | N/A | 288 | 160 | N/A | N/A | N/A | N/A | N/A | Sheep: 38.8%  Goats: 21.6% |
| Krametter‐Frötscher et al., 2007 | Austria | Ab-ELISA, VNT | N/A | N/A | 4931 (1448) | N/A | N/A | N/A | N/A | N/A | N/A | Sheep: 29.4% |
| Broaddus et al., 2007 | United States | VNT, VI, IHC | N/A | N/A | 10 (10) | N/A | N/A | N/A | N/A | N/A | N/A | Sheep: 100% |
| Wakeley et al., 2004 | UK | Ag-ELISA, RT-qPCR | Detected | N/A | Detected | N/A | N/A | N/A | N/A | N/A | N/A | Not estimated |
| Graham et al., 2001 | Ireland | VNT | N/A | N/A | 918 (14) | N/A | 660 (1) | N/A | N/A | N/A | N/A | Sheep: 1.5%;  Pig: 0.15% |
| Celedon et al., 2001 | Chile | VNT | N/A | N/A | 321 (60) | 322 (21) | N/A | N/A | N/A | 74 (8) Alpacas, 43 (6) Llamas | N/A | Sheep: 18.7%  Goat: 6.5%  Alpacas: 10.8%  Llamas: 14% |
| Tegtmeier et al., 2000 | Denmark | Ab-ELISA | N/A | N/A | Detected | Detected | N/A | N/A | N/A | N/A | N/A | Not estimated |
| **Goat** |  |  | N/A | N/A | N/A | N/A | N/A | N/A | N/A |  | N/A |  |
| Al-Mubarak et al., 2022 | Saudi Arabia | Ab-ELISA | N/A | N/A | N/A | 84 (3) | N/A | N/A | N/A | 906 (38) | N/A | Camel: 4.1%  Goat: 3.5% |
| Hidayat et al., 2021 | Indonesia | Ab-ELISA; RT-PCR | N/A | N/A | 26  ( 2) | 20  (2) | N/A | N/A | N/A | N/A | N/A | Sheep: 7.7%  Goat: 10% |
| Berg et al., 2019 | South Africa | Ab-ELISA, Sequencing | 364  (195) | N/A | N/A | 100  (0) | N/A | N/A | N/A | N/A | N/A | Cattle: 53.6%;  Goat: 0% |
| Decaro et al., 2017 | Italy | Ab- ELISA, RT-PCR, Sequencing | 44 farm positive | one farm positive | 21 flocks positive | 6 flocks positive | N/A | N/A | N/A | N/A | N/A | Not estimated |
| Shi et al., 2016 | China | Ab- ELISA, RT-PCR, Sequencing | N/A | N/A | 49 (6) | 49 (6) | N/A | N/A | N/A | N/A | N/A | Sheep: 12.2%  Goat:12.2% |
| Abdel-Latif et al., 2013 | Egypt | IHC, VI, RT-PCR, Sequencing | N/A | N/A | N/A | Detected | N/A | N/A | N/A | N/A | N/A | Not estimated |
| Safarpoor Dehkordi, 2011 | Iran | RT-PCR | 620 (127) | 372 (76) | 525 (74) | 442 (93) | N/A | N/A | N/A | 214 (32) | N/A | Cattle: 20.48%  Buffalo: 20.43%  Sheep: 14.09%  Goat: 21.04%  Camel: 14.95% |
| Czopowicz et al., 2011 | Poland | Ab-ELISA | N/A | N/A | N/A | 1060 (108) | N/A | N/A | N/A | N/A | N/A | Goat: 10.2% |
| Danuser et al., 2009 | Switzerland | Ab-ELISA, VNT | N/A | N/A | 5059 (815) | 503 (128) | N/A | N/A | N/A | 109 (5) | N/A | Sheep: 16.1%  Goats: 25.4%  Alpacas and Lamas :4.6% |
| Mishra et al., 2009 | India | Ab-ELISA, Ag-ELISA, VNT | N/A | N/A | 1077 | 1026 | N/A | N/A | N/A | N/A | N/A | Sheep: 23.4%  Goat: 16.9% |
| Yesilbag and Gungor, 2009 | Turkey | VNT, VI | N/A | N/A | 288 | 160 | N/A | N/A | N/A | N/A | N/A | Sheep: 38.8%  Goats: 21.6% |
| Mishra et al., 2007 | India | Ag-ELISA, Ab-ELISA, RT-PCR | N/A | N/A | - | 562 (2) | N/A | N/A | N/A | N/A | N/A | Goat: 0.35% |
| Krametter Froetscher et al., 2006 | Austria | Ab-ELISA,VNT | N/A | N/A | - | 549 (63) | N/A | N/A | N/A | N/A | N/A | Goat: 11.5% |
| Celedon et al., 2001 | Chile | VNT | N/A | N/A | 321 (60) | 322 (21) | N/A | N/A | N/A | 74 (8) Alpacas, 43 (6) Llamas | N/A | Sheep: 18.7%  Goat: 6.5%  Alpacas: 10.8%  Llamas: 14% |
| Tegtmeier et al., 2000 | Denmark | Ab-ELISA | N/A | N/A | Detected | Detected | N/A | N/A | N/A | - | N/A | Not estimated |
| **Pig** |  |  |  |  |  |  |  |  |  |  |  |  |
| Kennedy et al., 2024 | USA | VNT, VI | N/A | N/A | N/A | N/A | 945 (51) | N/A | N/A | N/A | N/A | Wild pig: 16.7% |
| Choe et al., 2022 | South Korea | Ab-ELISA, RT-PCR , Sequencing | N/A | N/A | N/A | N/A | 168 (54) | N/A | N/A | N/A | N/A | Pig herd: 32.14% |
| Nurain et al., 2020 | Malaysia | RT-PCR | N/A | N/A | N/A | N/A | Detected | N/A | N/A | N/A | N/A | Not estimated |
| Milićević et al., 2018 | Serbia | RT-PCR, Sequencing | N/A | N/A | N/A | N/A | 50 (4) | N/A | N/A | N/A | N/A | Pig: 8% |
| Gatto et al., 2018 | Brazil | Ab-ELISA, VNT | N/A | N/A | N/A | N/A | 33 (21) | N/A | N/A | N/A | N/A | Pig herd: 64% |
| Chakraborty et al., 2018 | India | Ab-ELISA, RT-PCR, RT-qPCR, Sequencing | N/A | N/A | N/A | N/A | 206 (10) | N/A | N/A | N/A | N/A | Pig: 4.86% |
| Rodrígue-Prieto et al., 2016 | Spain | Ab-ELISA, RT-qPCR | 180  (82) | N/A | N/A | N/A | 62  (0) | 267  (52) | N/A | N/A | N/A | Cattle: 45.5%,  Deer: 19.5%  Pig:0% |
| Deng et al., 2012 | China | Ab-ELISA, RT-PCR | N/A | N/A | N/A | N/A | 212 (50) | N/A | N/A | N/A | N/A | Pig: 23.6% |
| Loeffen et al., 2009 | Netherlands | Ag-ELISA,VNT | N/A | N/A | N/A | N/A | Sow: 6020 (616 ) herds;  Finishing pigs: 1890 (189 ) herds | N/A | N/A | N/A | N/A | Sows:  Herd level: 11%  Animal level: 2.5%  Finishing pigs:  Herd level: 3.2%  Animal level: 0.42% |
| Graham et al., 2001 | Ireland | VNT | N/A | N/A | 918 (14) | N/A | 660 (1) | N/A | N/A | N/A | N/A | Sheep: 1.5%;  Pig: 0.15% |
| **Deer** |  |  |  |  |  |  |  |  |  |  |  |  |
| Zhigailov et al., 2023 | Kazakhstan | Ab-ELISA, RT-PCR | 2477  (1965) | N/A | N/A | N/A | N/A | 21  (4) | N/A | N/A | N/A | Cattle: 79.3%,  Deer: 19.1% |
| Medina-Gudiño et al., 2022 | Mexico | RT-PCR , Sequencing | N/A | Detected | N/A | N/A | N/A | Detected | N/A | N/A | N/A | Not estimated |
| Huaman et al., 2020 | Australia | Ag and Ab-ELISA | N/A | N/A | N/A | N/A | N/A | Detected | N/A | N/A | N/A | Deer: 3% |
| Ricci et al., 2019 | Italy | Ag-ELISA, RT-PCR, Sequencing | Detected | N/A | Detected | N/A | N/A | Detected | N/A | N/A | N/A | Not estimated |
| Graham et al., 2017 | Ireland | Ab- ELISA | N/A | N/A | N/A | N/A | N/A | Detected | N/A | N/A | N/A | Not estimated |
| Rodrígue-Prieto et al., 2016 | Spain | Ab-ELISA, RT-qPCR | 180  (82) | N/A | N/A | N/A | 62  (0) | 267  (52) | N/A | N/A | N/A | Cattle: 45.5%,  Deer: 19.5%  Pig:0% |
| Kirchgessner et al., 2013 | United States | ELISA, PCR, ICH | N/A | N/A | N/A | N/A | N/A | 1069 (80) | N/A | N/A | N/A | Deer: 7% |
| Gao et al., 2011 | China | VI, TEM, Sequencing | N/A | N/A | N/A | N/A | N/A | Detected | N/A | N/A | N/A | Not estimated |
| Passler et al., 2008 | America | VNT, IHC | N/A | N/A | N/A | N/A | N/A | VNT: 165 (2)  ICH: 406 (1) | N/A | N/A | N/A | Deer ( VNT): 1.22 %; Deer ( ICH ): 0.25% |
| Chase et al., 2008 | America | IHC, RT-PCR | N/A | N/A | N/A | N/A | N/A | 2 (2) | N/A | N/A | N/A | Deer: 100% |
| Duncan et al., 2008 | America | IHC, RT-PCR | N/A | N/A | N/A | N/A | N/A | 5597 (2934 mule deer, 2516 elk, 141 white-taileddeer, and 6 moose) | N/A | N/A | N/A | Deer : 0.52% |
| Cantu et al., 2008 | Mexico | Ab-ELISA | N/A | N/A | N/A | N/A | N/A | 521 (331) | N/A | N/A | N/A | Deer: 63.5% |
| Wolf et al., 2008 | United States | Ab-ELISA | N/A | N/A | N/A | N/A | N/A | 114 (46) | N/A | N/A | N/A | Deer: 41% |
| Köppel  et al., 2007 | Switzerland | Ab-ELISA | N/A | N/A | N/A | N/A | N/A | 234 (4) | N/A | N/A | N/A | Deer: 1.7% |
| Dubay et al., 2006 | United States | Ab-ELISA | N/A | N/A | N/A | N/A | N/A | 128 (7) | N/A | N/A | N/A | Deer: 5.5% |
| Grøndahl et al., 2003 | Denmark | Ab-ELISA, IImT , VI, RT-PCR, Sequencing | N/A | N/A | N/A | N/A | N/A | 4 (3) | N/A | N/A | N/A | Deer: 75% |
| Lillehaug et al., 2003 | Norway | Ab-ELISA, VNT | N/A | N/A | N/A | N/A | N/A | Detected | N/A | N/A | N/A | Roe deer: 12.3% , Rein deer: 4.2%, Moose deer: 2.0% and Red deer: 1.1% |
| Jones et al., 2001 | Argentina | RT-PCR, Sequencing | N/A | N/A | N/A | N/A | N/A | Detected | N/A | N/A | N/A | Not estimated |
| Van Campen et al., 2001 | United States | RT-PCR | N/A | N/A | N/A | N/A | N/A | 124 (74) | N/A | N/A | N/A | Deer: 60% |
| Nielsen et al., 2000 | Denmark | Ag-ELISA, VNT | N/A | N/A | N/A | N/A | N/A | 476 (3) | N/A | N/A | N/A | Deer: 0.63% |
| **Bison** |  |  |  |  |  |  |  |  |  |  |  |  |
| Hause et al., 2021 | United States | VI, RT-PCR | N/A | N/A | N/A | N/A | N/A | - | 4 (2) | N/A | N/A | Bison: 50% |
| Salwa et al., 2007 | Poland | Ab-ELISA | N/A | N/A | N/A | N/A | N/A | **-** | 122 (36) | N/A | N/A | Bison: 29.5% |
| Sausker and Dyer, 2002 | United States | SNT | N/A | N/A | N/A | N/A | N/A | - | 226 (125) | N/A | N/A | Bison: 55.3% |
| **Yak** |  |  |  |  |  |  |  |  |  |  |  |  |
| Ma et al., 2016 | China | Ab-ELISA | N/A | N/A | N/A | N/A | N/A | N/A | N/A | N/A | 1584(595) | Yak: 37.6% |
| Ochirkhuu et al., 2016 | Mongolia | Ag-ELISA, VI, RT-PCR, IHC, Sequencing | 15.8 % | N/A | N/A | N/A | N/A | N/A | N/A | N/A | 20.0 % | Cattle:15.8 %  Yak: 20.0 % |
| Deng et al., 2015 | China | Ag-ELISA, Ab-ELISA, RT-PCR , Sequencing | Dairy cattle: (298/333), Beef cattle: (248/392), | 134 (26) | N/A | N/A | N/A | N/A | N/A | N/A | yaks: (236/520) | Dairy cattle: 89.49%, Beef cattle: 63.27% Water Buffalo : 19.40% ; Yak: 45.38% |
| Van Campen et al., 2013 | United States | Ab-ELISA, RT-PCR | N/A | N/A | N/A | N/A | N/A | N/A | N/A | N/A | Detected | Not estimates |
| Mishra et al., 2008 | India | Ag ELISA, RT-PCR | N/A | N/A | N/A | N/A | N/A | N/A | N/A | N/A | Detected | Not estimated |
| **Camelids** |  |  |  |  |  |  |  |  |  |  |  |  |
| Al-Mubarak et al., 2022 | Saudi Arabia | Ab-ELISA | N/A | N/A | N/A | 84 (3) | N/A | N/A | N/A | 906 (38) | N/A | Camel: 4.1%  Goat: 3.5% |
| Ataseven et al., 2022 | Turkey | Ab-ELISA, Ag-ELISA | N/A | N/A | N/A | N/A | N/A | N/A | N/A | Detected | N/A | Not estimated |
| Tesfaye et al., 2021 | Ethiopia | Ab-ELISA, Ag- ELISA | 219 (177) | N/A | N/A | N/A | N/A | N/A | N/A | 348 (8) | N/A | Cattle: 80.82%  Camel: 2.29% |
| Liu et al., 2021 | China | Ab-ELISA | N/A | N/A | N/A | N/A | N/A | N/A | N/A | 182 (6) | N/A | Alpacas: 3.25% |
| Dittmer et al., 2018 | New Zealand | Ab-ELISA, RT-PCR | N/A | N/A | N/A | N/A | N/A | N/A | N/A | 195 (4) | N/A | Alpacas: 2.1% |
| Saidi et al., 2018 | Algeria | Ag -ELISA, Ab-ELISA, | N/A | N/A | N/A | N/A | N/A | N/A | N/A | 111 (46) | N/A | Camel: 41.4% |
| Malek et al., 2017 | Egypt | Ab- ELISA | N/A | N/A | N/A | N/A | N/A | N/A | N/A | 92 (25) | N/A | Camel: 27.2% |
| Khalafalla et al., 2017 | Saudi Arabia | Ab- ELISA | N/A | N/A | N/A | N/A | N/A | N/A | N/A | 182 (53) | N/A | Camel: 29.1% |
| Stanitznig et al., 2016 | Austria | Ab- ELISA | N/A | N/A | N/A | N/A | N/A | N/A | N/A | 261 (1) | N/A | Alpacas: 0.2% |
| Al-Rubayie et al., 2016 | Iraq | Ab- ELISA | N/A | N/A | N/A | N/A | N/A | N/A | N/A | 88 (12**)** | N/A | Camel: 13.63% |
| Peter et al., 2015 | Nigeria | Ab-ELISA | N/A | N/A | N/A | N/A | N/A | N/A | N/A | 90 (28) | N/A | Camel:31.1% |
| Gao et al., 2013 | China | Ab-ELISA, VNT, RT-qPCR, Sequencing | N/A | N/A | N/A | N/A | N/A | N/A | N/A | 56 (17) | N/A | Camel: 30% |
| Safarpoor Dehkordi, 2011 | Iran | RT-PCR | 620 (127) | 372 (76) | 525 (74) | 442 (93) | N/A | N/A | N/A | 214 (32) | N/A | Cattle: 20.48%  Buffalo: 20.43%  Sheep: 14.09%  Goat: 21.04%  Camel: 14.95% |
| Raoofi et al., 2010 | Iran | SNT | N/A | N/A | N/A | N/A | N/A | N/A | N/A | 137 (27) | N/A | Camel: 19.7% |
| Turcitu et al., 2010 | Romania | RT-PCR, RT-qPCR | N/A | N/A | N/A | N/A | N/A | N/A | N/A | Detected | N/A | Not estimated |
| Intisar et al., 2010 | Sudan | Ab-ELISA, FAT, RT-PCR | N/A | N/A | N/A | N/A | N/A | N/A | N/A | 260 (220) | N/A | Camel: 84.6% |
| Topliff et al., 2009 | United States | SNT, RT-PCR | N/A | N/A | N/A | N/A | N/A | N/A | N/A | 63  (16) | N/A | Alpaca  Herds: 25.4% |
| Danuser et al., 2009 | Switzerland | Ab-ELISA, VNT | N/A | N/A | 5059 (815) | 503 (128) | N/A | N/A | N/A | 109 (5) | N/A | Sheep: 16.1%  Goats: 25.4%  Alpacas and Lamas :4.6% |
| Celedon et al., 2001 | Chile | VNT | N/A | N/A | 321 (60) | 322 (21) | N/A | N/A | N/A | 74 (8) Alpacas, 43 (6) Llamas | N/A | Sheep: 18.7%  Goat: 6.5%  Alpacas: 10.8%  Llamas: 14% |
| Foster et al., 2005 | United Kingdom (UK) | IHC, RT-PCR | N/A | N/A | N/A | N/A | N/A | N/A | N/A | 1(1) | N/A | Alpaca: 100% |
| Carman et al., 2005 | Canada | Ag-ELISA, VNT, RT-PCR | N/A | N/A | N/A | N/A | N/A | N/A | N/A | 13 (1) | N/A | Alpaca: 7.69% |
| Wentz et al., 2003 | United States | RT-PCR | N/A | N/A | N/A | N/A | N/A | N/A | N/A | 223 (2) | N/A | Llamas : 0.9% |
| Goyal et al., 2002 | United States | RT-PCR | N/A | N/A | N/A | N/A | N/A | N/A | N/A | 1 (1) | N/A | Alpaca: 100% |
| Belknap et al., 2000 | United States | VI, Immunoperoxidase staining, IFA | N/A | N/A | N/A | N/A | N/A | N/A | N/A | 174 (4) | N/A | Llamas: 2.3% |

N/A: Not applicable; Ag-ELISA: Antigen-capture enzyme linked immunosorbent assay; Ab-ELISA: Antibody-capture enzyme linked immunosorbent assay, RT-PCR: Reverse transcription polymerase chain reaction; RT-qPCR: Quantitative reverse transcription polymerase chain reaction; VI: Virus isolation; VNT: Virus neutralization test; SNT: Serum neutralization test; IHC: Immunohistochemistry; IFA: Immunofluorescence Assay; TEM: transmission electron microscope; Indirect immunoperoxidase test: IImT.

**References**

1. Birhanu W, Tesfaye A, Getachew Y, Negussie H. Seroprevalence of bovine viral diarrhea virus and detection of persistently infected (PI) animals in dairy farms of Holeta, central Ethiopia. Ethiopian Veterinary Journal. 2024 Mar 18;28(1):73-87.
2. Zhigailov AV, Perfilyeva YV, Ostapchuk YO, Kan SA, Lushova AV, Kuligin AV, Ivanova KR, Kuatbekova SA, Abdolla N, Naizabayeva DA, Maltseva ER. Molecular and serological survey of bovine viral diarrhea virus infection in cattle in Kazakhstan. Research in Veterinary Science. 2023 Sep 1;162:104965.
3. Hugues F, Cabezas I, Garigliany M, Rivas F, Casanova T, González EE, Sánchez O, Castillo R, Parra NC, Inostroza-Michael O, Moreno L. First report of bovine viral diarrhea virus subgenotypes 1d and 1e in southern Chile. Virology Journal. 2023 Sep 7;20(1):205.
4. Karimi O, Bitaraf Sani M, Bakhshesh M, Harofteh JZ, Poormirzayee H. Seroprevalence of bovine viral diarrhea virus antibodies and risk factors in dairy cattle from the central desert of Iran. Tropical Animal Health and Production. 2022 Jun;54(3):176.
5. Kučer N, Marković E, Prpić J, Rudan D, Jemeršić L, Hajek Z, Škaro K, Pavlak M, Rudan N. Epidemiology of bovine viral diarrhea virus infection on a dairy farm-clinical signs, seroprevalence, virus detection and genotyping. Veterinarski arhiv. 2022 May 19;92(2):119-26.
6. Ahmad A, Awan FN, Rabbani M, Mushtaq MH. Molecular characterization and phylogeny of bovine viral diarrhea virus isolated from persistently infected exotic cattle in Punjab, Pakistan. Pakistan Journal of Agricultural Sciences. 2022 Jul 1;59(4).
7. Antos A, Rola J, Bednarski M, Krzysiak MK, Kęsik-Maliszewska J, Larska M. Is contamination of bovine-sourced material with bovine viral diarrhea virus still a problem in countries with ongoing eradication campaigns?. Annals of Animal Science. 2021 Jan 1;21(1):173-92.
8. Spetter MJ, Uriarte EL, Verna AE, Leunda MR, Pereyra SB, Odeón AC, Altamiranda EA. Genomic diversity and phylodynamic of bovine viral diarrhea virus in Argentina. Infection, Genetics and Evolution. 2021 Dec 1;96:105089.
9. Tesfaye A, Omer A, Hussein A, Garoma A, Guyassa C, Paeshuyse J, Tolera TS. Seroprevalence of bovine viral diarrhea virus in local Borana cattle breed and camels (Camelus dromedarius) in Ethiopia. Veterinary Medicine: Research and Reports. 2021 Jun 8:141-8.
10. VanLeeuwen J, Muraya J, Gitau G, Makau D, Crane B, McKenna S, Wichtel J. Seroprevalence and risk factors of neospora caninum and bovine viral diarrhoea virus in smallholder dairy cattle in Kenya. East African Journal of Science, Technology and Innovation. 2021 Dec 17;3(1).
11. Toplak I, Hostnik P, Černe D, Mrkun J, Starič J. The principles of the voluntary programme for the control and elimination of bovine viral diarrhoea virus (BVDV) from infected herds in Slovenia. Frontiers in veterinary science. 2021 Jul 19;8:676473.
12. Caffarena RD, Casaux ML, Schild CO, Fraga M, Castells M, Colina R, Maya L, Corbellini LG, Riet-Correa F, Giannitti F. Causes of neonatal calf diarrhea and mortality in pasture-based dairy herds in Uruguay: A farm-matched case-control study. Brazilian Journal of Microbiology. 2021 Jun;52(2):977-88.
13. Guo T, Zhang J, Chen X, Wei X, Wu C, Cui Q, Hao Y. Investigation of viral pathogens in cattle with bovine respiratory disease complex in Inner Mongolia, China. Microbial Pathogenesis. 2021 Apr 1;153:104594.
14. Chang L, Qi Y, Liu D, Du Q, Zhao X, Tong D. Molecular detection and genotyping of bovine viral diarrhea virus in Western China. BMC veterinary research. 2021 Dec;17:1-7.
15. Deng M, Chen N, Guidarini C, Xu Z, Zhang J, Cai L, Yuan S, Sun Y, Metcalfe L. Prevalence and genetic diversity of bovine viral diarrhea virus in dairy herds of China. Veterinary microbiology. 2020 Mar 1;242:108565.
16. Hou P, Zhao G, Wang H, He H. Prevalence of bovine viral diarrhea virus in dairy cattle herds in eastern China. Tropical animal health and production. 2019 May 1;51:791-8.
17. McMorrow C, Gunn AJ, Khalfan S, Hernandez-Jover M, Brookes VJ. Veterinarians’ knowledge, attitudes and practices associated with bovine viral diarrhoea virus control and prevention in South-East Australia. Animals. 2020 Sep 11;10(9):1630.
18. Rypuła K, Płoneczka-Janeczko K, Czopowicz M, Klimowicz-Bodys MD, Shabunin S, Siegwalt G. Occurrence of BVDV infection and the presence of potential risk factors in dairy cattle herds in Poland. Animals. 2020 Jan 31;10(2):230.
19. Guidoum KA, Benallou B, Pailler L, Espunyes J, Napp S, Cabezón O. Ruminant pestiviruses in North Africa. Preventive veterinary medicine. 2020 Nov 1;184:105156.
20. Akagami M, Seki S, Kashima Y, Yamashita K, Oya S, Fujii Y, Takayasu M, Yaguchi Y, Suzuki A, Ono Y, Ouchi Y. Risk factors associated with the within-farm transmission of bovine viral diarrhea virus and the incidence of persistently infected cattle on dairy farms from Ibaraki prefecture of Japan. Research in veterinary science. 2020 Apr 1;129:187-92.
21. Nugroho W, Reichel MP, Ruff N, Gazali AM, Sakke IS. Infection with Bovine Viral Diarrhea Virus in cattle in Southern Papua, Indonesia. Acta Tropica. 2020 Dec 1;212:105712.
22. Noaman V, Nabinejad AR. Seroprevalence and risk factors assessment of the three main infectious agents associated with abortion in dairy cattle in Isfahan province, Iran. Tropical animal health and production. 2020 Jul;52:2001-9.
23. Berg M, Ramabu SS, Wensman JJ, Lysholm S. First-time detection of bovine viral diarrhoea virus, BVDV-1, in cattle in Botswana. Onderstepoort Journal of Veterinary Research. 2019 Jan 1;86(1):1-7.
24. Kim Y, Kim Y, Lee SY, Lee KK, Lee KH, Song JC, Oem JK. Identification of Korean native cattle persistently infected with BVDV using Ear-notch method. Korean Journal of Veterinary Service. 2019; 42(2):117-20.
25. Agah MA, Notsu K, El-Khaiat HM, Arikawa G, Kubo M, Mitoma S, Okabayashi T, Mekata H, Elhanafy E, El Daous H, Mai TN. Slaughterhouse survey for detection of bovine viral diarrhea infection among beef cattle in Kyushu, Japan. Journal of Veterinary Medical Science. 2019;81(10):1450-4.
26. Thapa A, Acharya MP, Raut R, Rimal S. Seroprevalence and Risk Factors of Bovine Viral Diarrhea in Improved Cattle of Chitwan, Nawalpur and Rupandehi Districts of Nepal. Nepalese Veterinary Journal. 2019 Dec 1;36:93-7.
27. Tadesse T, Deneke Y, Deresa B. Seroprevalence of bovine viral diarrhea virus and its potential risk factors in dairy cattle of Jimma town, southwestern Ethiopia. J Dairy, Vet Anim Res. 2019;8(1):11-7.
28. Ricci S, Bartolini S, Morandi F, Cuteri V, Preziuso S. Genotyping of Pestivirus A (Bovine Viral Diarrhea Virus 1) detected in faeces and in other specimens of domestic and wild ruminants at the wildlife-livestock interface. Veterinary microbiology. 2019 Aug 1;235:180-7.
29. Ryu JH, Choi KS. Genetic analysis of bovine viral diarrhea virus in pre-weaned native Korean calves. Tropical animal health and production. 2019 Sep 1;51:2085-90.
30. Quintero Barbosa, Juan, Adriana P. Corredor Figueroa, Sandra S. Salas, Hugo Camargo, Alfredo Sanchéz, Julio Tobón, Diego Ortiz, Eric Schachtebeck, and Maria Fernanda Gutierrez. 2019. 'High prevalence of persistently infected animals from bovine viral diarrhea in Colombian cattle', BMC veterinary research, 15: 23.
31. Bulut H, Sozdutmaz I, Pestildot over˜ Z, Abayli H, Sait A, Cevik A. High prevalence of bovine viral diarrhea virus-1 in sheep abortion samples with pestivirus infection in Turkey.
32. Kumar SK, Palanivel KM, Sukumar K, Ronald BS, Selvaraju G, Ponnudurai G. Herd-level risk factors for bovine viral diarrhea infection in cattle of Tamil Nadu. Tropical animal health and production. 2018 Apr;50:793-9.
33. Han DG, Ryu JH, Park J, Choi KS. Identification of a new bovine viral diarrhea virus subtype in the Republic of Korea. BMC veterinary research. 2018 Dec;14:1-7.
34. Olmo L, Dye MT, Reichel MP, Young JR, Nampanya S, Khounsy S, Thomson PC, Windsor PA, Bush RD. Investigation of infectious reproductive pathogens of large ruminants: Are neosporosis, brucellosis, leptospirosis and BVDV of relevance in Lao PDR?. Acta tropica. 2018 Jan 1;177:118-26.
35. Reichel MP, Lanyon SR, Hill FI. Perspectives on current challenges and opportunities for bovine viral diarrhoea virus eradication in Australia and New Zealand. Pathogens. 2018 Jan 22;7(1):14.
36. Hasan SD, Alsaad KM. Bovine viral diarrhea and persistently infection of cattle at Nineveh province, Iraq. Basrah J Vet Res. 2018;17:14-32.
37. Stalder H, Bachofen C, Schweizer M, Zanoni R, Sauerländer D, Peterhans E. Traces of history conserved over 600 years in the geographic distribution of genetic variants of an RNA virus: Bovine viral diarrhea virus in Switzerland. PLoS One. 2018 Dec 5;13(12):e0207604.
38. Viana RB, Kzam AD, Monteiro BM, Campello CC, de Moura Sousa E, De Souza DC, Okuda LH, Pituco EM, Ribeiro Filho JD. Sensitivity and specificity of indirect ELISA for the detection of antibody titers against BVDV from beef cattle raised in Pará State. Semina: Ciências Agrárias. 2017;38(5):3049-58.
39. Decaro N, Lucente MS, Lanave G, Gargano P, Larocca V, Losurdo M, Ciambrone L, Marino PA, Parisi A, Casalinuovo F, Buonavoglia C. Evidence for circulation of bovine viral diarrhoea virus type 2c in ruminants in Southern Italy. Transboundary and emerging diseases. 2017 Dec; 64(6):1935-44.
40. Wolff C, Boqvist S, Ståhl K, Masembe C, Sternberg-Lewerin S. Biosecurity aspects of cattle production in Western Uganda, and associations with seroprevalence of brucellosis, salmonellosis and bovine viral diarrhoea. BMC veterinary research. 2017 Dec;13(1):1-6.
41. Wernike K, Schirrmeier H, Strebelow HG, Beer M. Eradication of bovine viral diarrhea virus in Germany—Diversity of subtypes and detection of live-vaccine viruses. Veterinary microbiology. 2017 Sep 1;208:25-9.
42. Uddin MA, Ahasan AL, Islam K, Islam MZ, Mahmood A, Islam A, Islam KM, Ahad A. Seroprevalence of bovine viral diarrhea virus in crossbred dairy cattle in Bangladesh. Veterinary world. 2017 Aug;10(8):906.
43. Kaveh AA, Merat E, Samani S, Danandeh R, Soltannezhad S. Infectious causes of bovine abortion in Qazvin Province, Iran. Archives of Razi Institute. 2017 Dec 1;72(4):225-30.
44. Rodríguez-Prieto V, Kukielka D, Rivera-Arroyo B, Martínez-López B, de las Heras AI, Sánchez-Vizcaíno JM, Vicente J. Evidence of shared bovine viral diarrhea infections between red deer and extensively raised cattle in south-central Spain. BMC Veterinary Research. 2016 Dec;12:1-1.
45. Daves L, Yimer N, Arshad SS, Sarsaifi K, Omar M, Yusoff R, Haron A, Abdullah F. Seroprevalence of bovine viral diarrhea virus (BVDV) infection and associated risk factors in cattle in Selangor, Malaysia. Vet. Med. Open J. 2016;1:22-8.
46. Hay KE, Ambrose RC, Morton JM, Horwood PF, Gravel JL, Waldron S, Commins MA, Fowler EV, Clements AC, Barnes TS, Mahony TJ. Effects of exposure to bovine viral diarrhoea virus 1 on risk of bovine respiratory disease in Australian feedlot cattle. Preventive Veterinary Medicine. 2016 Apr 1;126:159-69.
47. Segura-Correa JC, Zapata-Campos CC, Jasso-Obregón JO, Martinez-Burnes J, López-Zavala R. Seroprevalence and risk factors associated with bovine herpesvirus 1 and bovine viral diarrhea virus in North-Eastern Mexico. Open veterinary journal. 2016 Aug 26;6(2):143-9.
48. Evans CA, Cockcroft PD, Reichel MP. Antibodies to bovine viral diarrhoea virus (BVDV) in water buffalo (Bubalus bubalis) and cattle from the Northern Territory of Australia. Australian Veterinary Journal. 2016 Nov;94(11):423-6.
49. Glotov AG, Glotova TI, Nefedchenko AV, Koteneva SV. Genetic diversity and distribution of bovine pestiviruses (Flaviviridae: Pestivirus) in the world and in the Russian Federation. Problems of Virology. 2022 Mar 15;67(1):18-26.
50. Ochirkhuu N, Konnai S, Odbileg R, Odzaya B, Gansukh S, Murata S, Ohashi K. Molecular detection and characterization of bovine viral diarrhea virus in Mongolian cattle and yaks. Archives of virology. 2016 Aug;161:2279-83.
51. Bello SM, Daneji AI, Chafe UM, Abubakar MB, Jibril AH, Festus A. Detection of antibodies to bovine viral diarrhea virus in cattle presented for slaughter at Sokoto metropolitan abattoir, Nigeria. Journal of Veterinary Medicine and Animal Health. 2016 Feb 29;8(2):11-4.
52. Nilnont T, Aiumlamai S, Kanistanont K, Inchaisri C, Kampa J. Bovine viral diarrhea virus (BVDV) infection in dairy cattle herds in northeast Thailand. Tropical animal health and production. 2016 Aug;48:1201-8.
53. Sayers RG, Byrne N, O'Doherty E, Arkins S. Prevalence of exposure to bovine viral diarrhoea virus (BVDV) and bovine herpesvirus-1 (BoHV-1) in Irish dairy herds. Research in Veterinary Science. 2015 Jun 1;100:21-30.
54. Kulangara V, Joseph A, Thrithamarassery N, Sivasailam A, Kalappurackal L, Mattappillil S, Syam R, Mapranath S. Epidemiology of bovine viral diarrhoea among tropical small holder dairy units in Kerala, India. Tropical animal health and production. 2015 Mar;47:575-9.
55. Soltan MA, Wilkes RP, Elsheery MN, Elhaig MM, Riley MC, Kennedy MA. Circulation of bovine viral diarrhea virus–1 (BVDV-1) in dairy cattle and buffalo farms in Ismailia Province, Egypt. The Journal of infection in developing countries. 2015 Dec 30;9(12):1331-7.
56. Saeed IK, Ali YH, Taha KM, Mohammed NE, Nouri YM, Mohammed BA, Mohammed OI, Elmagbool SB, Elghazali F. First report of Bovine Viral Diarrhea Virus antigen from pneumonic cattle in Sudan. Journal of Advanced Veterinary and Animal Research. 2015 Jun 1;2(2):153-7.
57. Deng, M.; Ji, S.; Fei, W.;.Raza, S.; He, C.; Chen, Y.; Chen, H.; Guo, A. Prevalence study and genetic typing of bovine viral diarrhea virus (BVDV) in four bovine species in China. PloS one. 2015, 10(4), e0121718.
58. Stegniy B, Gerilovych A, Vilcek S, Peterhans E, Kucheriavenko R, Stegniy M, Goraichuk I, Bolotin V, Solodiankin O. Molecular epidemiology issues of BVDV infection in the Eastern Ukraine. Agricultural Science and Practice. 2014 Apr 15;1(1):37-41.
59. Mahmoud MA, Allam AM. Seroprevalence of bovine viral diarrhea virus (BVDV), bovine herpes virus type 1 (BHV-1), parainfluenza type 3 virus (PI-3V) and bovine respiratory syncytial virus (BRSV) among non vaccinated cattle. Global Veterinaria. 2013 Apr 24;10(3):348-53.
60. Kuta, A., M. P. Polak, M. Larska, and J. F. Żmudziński. 2013. 'Predominance of bovine viral diarrhea virus 1b and 1d subtypes during eight years of survey in Poland', Veterinary microbiology, 166: 639-44.
61. Aniță D, ANIȚĂ A, Savuța G. Prevalence of Antibodies to BVDV and BHV1 in Dairy Herds in Southeastern Region of Romania. Lucrari Stiintifice-Universitatea de Stiinte Agricole a Banatului Timisoara, Medicina Veterinara. 2013;46(3):5-9.
62. Alsaad KM, Al-Obaidi QT, Hassan SD. Clinical, haematological and coagulation studies of bovine viral diarrhoea in local Iraqi calves. Bulgarian Journal of Veterinary Medicine. 2012 Mar 1;15(1).
63. Sharifzadeh A, Doosti A, Dehkordi PG. Reverse Transcriptase PCR Assay for Detection of Bovine viral diarrhea virus (BVDV) Infection in Iranian Bull’s Semen Samples. Middle-East Journal of Scientific Research. 2011 Nov 30;9(1):132-9.
64. Safarpoor Dehkordi F. Prevalence study of Bovine viral diarrhea virus by evaluation of antigen capture ELISA and RT-PCR assay in Bovine, Ovine, Caprine, Buffalo and Camel aborted fetuses in Iran. AMB express. 2011 Dec;1:1-6.
65. Handel IG, Willoughby K, Land F, Koterwas B, Morgan KL, Tanya VN, Bronsvoort BM. Seroepidemiology of bovine viral diarrhoea virus (BVDV) in the Adamawa region of Cameroon and use of the SPOT test to identify herds with PI calves. PloS one. 2011 Jul 6;6(7):e21620.
66. Friedgut O, Rotenberg D, Brenner J, Yehuda S, Paz R, Alpert N, Ram A, Yadin H, Grummer B. Description of the first acute bovine diarrhea virus-2 outbreak in Israel. The Veterinary Journal. 2011 Jul 1;189(1):108-10.
67. Ståhl K, Benito A, Felmer R, Zuñiga J, Reinhardt G, Rivera H, Baule C, Moreno-López J. Genetic diversity of bovine viral diarrhoea virus (BVDV) from Peru and Chile. Pesquisa Veterinária Brasileira. 2009;29:41-4.
68. Talafha AQ, Hirche SM, Ababneh MM, Al-Majali AM, Ababneh MM. Prevalence and risk factors associated with bovine viral diarrhea virus infection in dairy herds in Jordan. Tropical animal health and production. 2009 Apr;41:499-506.
69. Guarino H, Nunez A, Repiso MV, Gil A, Dargatz DA. Prevalence of serum antibodies to bovine herpesvirus-1 and bovine viral diarrhea virus in beef cattle in Uruguay. Preventive veterinary medicine. 2008 Jun 15;85(1-2):34-40.
70. NOVÁČKOVÁ M, Jacková A, Kolesárová M, VILČEK Š. Genetic analysis of a bovine viral diarrhea virus 2 isolate from Slovakia. Acta virologica. 2008;52:161-6.
71. Booker CW, Abutarbush SM, Morley PS, Guichon PT, Wildman BK, Jim GK, Schunicht OC, Pittman TJ, Perrett T, Ellis JA, Appleyard G. The effect of bovine viral diarrhea virus infections on health and performance of feedlot cattle. The Canadian Veterinary Journal. 2008 Mar;49(3):253.
72. Duong MC, Alenius S, Huong LT, Björkman C. Prevalence of Neospora caninum and bovine viral diarrhoea virus in dairy cows in Southern Vietnam. The Veterinary Journal. 2008 Mar 1;175(3):390-4.
73. O'Connor AM, Reed MC, Denagamage TN, Yoon KJ, Sorden SD, Cooper VL. Prevalence of calves persistently infected with bovine viral diarrhea virus in beef cow-calf herds enrolled in a voluntary screening project. Journal of the American Veterinary Medical Association. 2007 Jun 1;230(11):1691-6.
74. Scott, H.M., Sorensen, O., Wu, J.T., Chow, E.Y., Manninen, K. and VanLeeuwen, J.A., 2006. Seroprevalence of Mycobacterium avium subspecies paratuberculosis, Neospora caninum, Bovine leukemia virus, and Bovine viral diarrhea virus infection among dairy cattle and herds in Alberta and agroecological risk factors associated with seropositivity. The Canadian veterinary journal, 47(10), p.981.
75. Billinis C, Leontides L, Amiridis GS, Spyrou V, Kostoulas P, Sofia M. Prevalence of BVDV infection in Greek dairy herds. Preventive veterinary medicine. 2005 Nov 15;72(1-2):75-9.
76. Uttenthal Å, Stadejek T, Nylin B. Genetic diversity of bovine viral diarrhoea viruses (BVDV) in Denmark during a 10‐year eradication period. Apmis. 2005 Aug;113(7‐8):536-41.
77. Wakeley PR, Turner JL, Ibata G, King DP, Sandvik T, Howard P, Drew TW. Characterisation of a type 2 bovine viral diarrhoea virus isolated from cattle in the UK. Veterinary microbiology. 2004 Aug 19;102(1-2):19-24.
78. Fulton RW, Ridpath JF, Saliki JT, Briggs RE, Confer AW, Burge LJ, Purdy CW, Loan RW, Duff GC, Payton ME. Bovine viral diarrhea virus (BVDV) 1b: predominant BVDV subtype in calves with respiratory disease. Canadian Journal of Veterinary Research. 2002 Jul;66(3):181.
79. Deregt D, Carman PS, Clark RM, Burton KM, Olson WO, Gilbert SA. A comparison of polymerase chain reaction with and without RNA extraction and virus isolation for detection of bovine viral diarrhea virus in young calves. Journal of veterinary diagnostic investigation. 2002 Sep;14(5):433-7.
80. Rossmanith W, Vilček Š, Wenzl H, Rossmanith E, Loitsch A, Durkovic B, Strojny L, Paton DJ. Improved antigen and nucleic acid detection in a bovine virus diarrhoea eradication program. Veterinary Microbiology. 2001 Aug 8;81(3):207-18.
81. Mainar-Jaime RC, Berzal-Herranz B, Arias P, Rojo-Vázquez FA. Epidemiological pattern and risk factors associated with bovine viral-diarrhoea virus (BVDV) infection in a non-vaccinated dairy-cattle population from the Asturias region of Spain. Preventive veterinary medicine. 2001 Nov 2;52(1):63-73.
82. Rush DM, Thurmond MC, Muñoz-Zanzi CA, Hietala SK. Descriptive epidemiology of postnatal bovine viral diarrhea virus infection in intensively managed dairy heifers. Journal of the American Veterinary Medical Association. 2001 Nov 15;219(10):1426-31.
83. Beaudeau F, Belloc C, Seegers H, Assie S, Sellal E, Joly A. Evaluation of a blocking ELISA for the detection of bovine viral diarrhoea virus (BVDV) antibodies in serum and milk. Veterinary Microbiology. 2001 Jun 22;80(4):329-37.
84. Haines DM, Martin KM, Clark EG, Jim GK, Janzen ED. The immunohistochemical detection of Mycoplasma bovis and bovine viral diarrhea virus in tissues of feedlot cattle with chronic, unresponsive respiratory disease and/or arthritis. The Canadian Veterinary Journal. 2001 Nov;42(11):857.
85. Pastrana ME, Carrascal-Triana E, Ramos MD, Ortega DO. Seroprevalence and associated factors of viral agents of the bovine respiratory disease complex in buffaloes of Colombia. Ciência Rural. 2022 Jan 5;52.
86. Paixão SF, Fritzen JT, Crespo SE, de Moraes Pereira H, Alfieri AF, Alfieri AA. Bovine viral diarrhea virus subgenotype 1b in water buffalos (Bubalus bubalis) from Brazil. Tropical animal health and production. 2018a Dec;50(8):1947-50.
87. Paixão SF, Fritzen JT, Alfieri AF, Alfieri AA. Virus neutralization technique as a tool to evaluate the virological profile for bovine viral diarrhea virus infection in dairy water buffalo (Bubalus bubalis) herds. Tropical animal health and production. 2018b Apr;50:911-4.
88. Pecora A, Pérez Aguirreburualde MS, Malacari DA, Zabal O, Sala JM, Konrad JL, Caspe SG, Bauermann F, Ridpath J, Dus Santos MJ. Serologic evidence of HoBi-like virus circulation in Argentinean water buffalo. Journal of Veterinary Diagnostic Investigation. 2017 Nov;29(6):926-9.
89. Martucciello A, De Mia GM, Giammarioli M, De Donato I, Iovane G, Galiero G. Detection of Bovine viral diarrhea virus from three water buffalo fetuses (Bubalus bubalis) in southern Italy. Journal of veterinary diagnostic investigation. 2009 Jan;21(1):137-40.
90. Ahmed WM, Zaher KS. A field contribution on the relation between reproductive disorders and bovine viral diarrhea virus infection in buffalo-cows. American-Eurasian J Agric & Environ Sci. 2008 Nov 19;3(5):736-42.
91. Mishra N, Dubey R, Galav V, Tosh C, Rajukumar K, Pitale SS, Pradhan HK. Identification of bovine viral diarrhoea virus type 1 in Indian buffaloes and their genetic relationship with cattle strains in 5’UTR. Current Science. 2007 Jul 10:97-100.
92. Hidayat W, Wuryastuty H, Wasito R. Detection of Pestivirus in small ruminants in Central Java, Indonesia. Veterinary World. 2021 Apr;14(4):996.
93. Evans CA, Han JH, Weston JF, Heuer C, Gates MC. Serological evidence for exposure to bovine viral diarrhoea virus in sheep co-grazed with beef cattle in New Zealand. New Zealand veterinary journal. 2020 Jul 3;68(4):238-41.
94. Feknous N, Hanon JB, Tignon M, Khaled H, Bouyoucef A, Cay B. Seroprevalence of border disease virus and other pestiviruses in sheep in Algeria and associated risk factors. BMC veterinary research. 2018 Dec;14:1-1.
95. Silveira S, Falkenberg SM, Elderbrook MJ, Sondgeroth KS, Dassanayake RP, Neill JD, Ridpath JF, Canal CW. Serological survey for antibodies against pestiviruses in Wyoming domestic sheep. Veterinary microbiology. 2018 Jun 1;219:96-9.
96. Shi H, Kan Y, Yao L, Leng C, Tang Q, Ji J, Sun S. Identification of natural infections in sheep/goats with HoBi‐like pestiviruses in China. Transboundary and emerging diseases. 2016 Oct;63(5):480-4.
97. Danuser R, Vogt HR, Kaufmann T, Peterhans E, Zanoni R. Seroprevalence and characterization of pestivirus infections in small ruminants and new world camelids in Switzerland. Schweizer Archiv für Tierheilkunde. 2009 Mar 1;151(3):109-17.
98. Mishra N, Rajukumar K, Tiwari A, Nema RK, Behera SP, Satav JS, Dubey SC. Prevalence of Bovine viral diarrhoea virus (BVDV) antibodies among sheep and goats in India. Tropical Animal Health and Production. 2009 Oct;41:1231-9.
99. Julia S, Craig MI, Jiménez LS, Pinto GB, Weber EL. First report of BVDV circulation in sheep in Argentina. Preventive Veterinary Medicine. 2009 Aug 1;90(3-4):274-7.
100. Yeşilbağ K, Güngör B. Antibody prevalence against respiratory viruses in sheep and goats in North-Western Turkey. Tropical animal health and production. 2009 Apr;41:421-5.
101. Krametter‐Frötscher R, Loitsch A, Kohler H, Schleiner A, Schiefer P, Möstl K, Golja F, Baumgartner W. Serological survey for antibodies against pestiviruses in sheep in Austria. Veterinary Record. 2007 May;160(21):726-30.
102. Broaddus CC, Holyoak GR, Dawson L, Step DL, Funk RA, Kapil S. Transmission of bovine viral diarrhea virus to adult goats from persistently infected cattle. Journal of veterinary diagnostic investigation. 2007 Sep;19(5):545-8.
103. Graham DA, Calvert V, German A, McCullough SJ. Pestiviral infections in sheep and pigs in Northern Ireland. Veterinary Record. 2001 Jan;148(3):69-72.
104. Celedon, M., A. Sandoval, J. Droguett, R. Calfio, L. Ascencio, J. Pizarro, and C. Navarro. "Survey for antibodies to pestivirus and herpesvirus in sheep, goats, alpacas (Lama pacos), llamas (Lama glama), guanacos (Lama guanicoe) and vicuña (Vicugna vicugna) from Chile." (2001): 165-172.
105. Tegtmeier C, Stryhn H, Uttenthal Å, Kjeldsen AM, Nielsen TK. Seroprevalence of border disease in Danish sheep and goat herds. Acta Veterinaria Scandinavica. 2000;41(4):339.
106. Al-Mubarak AI, Hussen J, Kandeel M, Al-Kubati AA, Falemban B, Skeikh A, Hemida MG. Risk-associated factors associated with the bovine viral diarrhea virus in dromedary camels, sheep, and goats in abattoir surveillance and semi-closed herd system. Veterinary World. 2022 Aug;15(8):1924.
107. Abdel-Latif A, Goyal S, Chander Y, Abdel-Moneim A, Tamam S, Madbouly H. Isolation and molecular characterisation of a pestivirus from goats in Egypt. Acta Veterinaria Hungarica. 2013 Jun 1;61(2):270-80.
108. Czopowicz M, Kaba J, Schirrmeier H, Bagnicka E, Szaluś-Jordanow O, Nowicki M, Witkowski L, Frymus T. Serological evidence for BVDV-1 infection in goats in Poland. Acta Veterinaria Hungarica. 2011 Sep 1;59(3):399-404.
109. Mishra N, Dubey R, Rajukumar K, Tosh C, Tiwari A, Pitale SS, Pradhan HK. Genetic and antigenic characterization of bovine viral diarrhea virus type 2 isolated from Indian goats (Capra hircus). Veterinary microbiology. 2007 Oct 6;124(3-4):340-7.
110. Kennedy SM, Passler T, Ditchkoff SS, Brown VR, Raithel GW, Chamorro MF, Walz PH, Kyriakis CS, Falkenberg SM. Seroprevalence of Bovine Viral Diarrhea Virus in Wild Pigs (Sus scrofa) in 17 States in the USA. Journal of Wildlife Diseases. 2024 May 16.
111. Choe S, Lim SI, Park GN, Song S, Shin J, Kim KS, Hyun BH, Kim JH, An DJ. Prevalence of Bovine Viral Diarrhea Virus Infections in Pigs on Jeju Island, South Korea, from 2009–2019 and Experimental Infection of Pigs with BVDV Strains Isolated from Cattle. Veterinary Sciences. 2022 Mar 21;9(3):146.
112. Nurain, S., D. Norlina, D. Roshaslinda, and H. Roslina. "Detection of Bovine viral diarrhoea virus in swine from Malaysia. Malaysian journal of veterinary research " (2020): 39-45.
113. Milićević V, Maksimović-Zorić J, Veljović L, Kureljušić B, Savić B, Cvetojević Đ, Jezdimirović N, Radosavljević V. Bovine viral diarrhea virus infection in wild boar. Research in veterinary science. 2018 Aug 1;119:76-8.
114. Gatto IR, Linhares DC, de Souza Almeida HM, Mathias LA, de Medeiros AS, Poljak Z, Samara SI, de Oliveira LG. Description of risk factors associated with the detection of BVDV antibodies in Brazilian pig herds. Tropical animal health and production. 2018 Apr;50:773-8.
115. Chakraborty AK, Mukherjee P, Karam A, Das S, Barkalita L, Puro K, Sanjukta R, Ghatak S, Sakuntala I, Laha RG, Borah P. Suppl-2, M7: Evidence of BVDV in Pigs from North Eastern Part of India-Genetic Profiling and Characterisation. The Open Virology Journal. 2018;12:110.
116. Rodríguez-Prieto V, Kukielka D, Rivera-Arroyo B, Martínez-López B, de las Heras AI, Sánchez-Vizcaíno JM, Vicente J. Evidence of shared bovine viral diarrhea infections between red deer and extensively raised cattle in south-central Spain. BMC Veterinary Research. 2016 Dec;12:1-1.
117. Deng Y, Sun CQ, Cao SJ, Lin T, Yuan SS, Zhang HB, Zhai SL, Huang L, Shan TL, Zheng H, Wen XT. High prevalence of bovine viral diarrhea virus 1 in Chinese swine herds. Veterinary microbiology. 2012 Oct 12;159(3-4):490-3.
118. Loeffen WL, Van Beuningen A, Quak S, Elbers AR. Seroprevalence and risk factors for the presence of ruminant pestiviruses in the Dutch swine population. Veterinary Microbiology. 2009 May 12;136(3-4):240-5.
119. Huaman JL, Pacioni C, Forsyth DM, Pople A, Hampton JO, Carvalho TG, Helbig KJ. Serosurveillance and molecular investigation of wild deer in Australia reveals seroprevalence of Pestivirus infection. Viruses. 2020 Jul 13;12(7):752.
120. Graham DA, Gallagher C, Carden RF, Lozano JM, Moriarty J, O’Neill R. A survey of free-ranging deer in Ireland for serological evidence of exposure to bovine viral diarrhoea virus, bovine herpes virus-1, bluetongue virus and Schmallenberg virus. Irish veterinary journal. 2017 Dec;70:1-1.
121. Kirchgessner MS, Dubovi EJ, Whipps CM. Spatial point pattern analyses of Bovine viral diarrhea virus infection in domestic livestock herds and concomitant seroprevalence in wild white-tailed deer (Odocoileus virginianus) in New York State, USA. Journal of veterinary diagnostic investigation. 2013 Mar;25(2):226-33.
122. Gao Y, Wang S, Du R, Wang Q, Sun C, Wang N, Zhang P, Zhang L. Isolation and identification of a bovine viral diarrhea virus from sika deer in china. Virology journal. 2011 Dec;8(1):1-6.
123. Passler T, Walz PH, Ditchkoff SS, Walz HL, Givens MD, Brock KV. Evaluation of hunter-harvested white-tailed deer for evidence of bovine viral diarrhea virus infection in Alabama. Journal of veterinary diagnostic investigation. 2008 Jan;20(1):79-82.
124. Chase CC, Braun LJ, Leslie-Steen P, Graham T, Miskimins D, Ridpath JF. Bovine viral diarrhea virus multiorgan infection in two white-tailed deer in southeastern South Dakota. Journal of wildlife diseases. 2008 Jul 1;44(3):753-9.
125. Duncan C, Van Campen H, Soto S, LeVan IK, Baeten LA, Miller MW. Persistent Bovine viral diarrhea virus infection in wild cervids of Colorado. Journal of veterinary diagnostic investigation. 2008 Sep;20(5):650-3.
126. Cantu A, Ortega-S JA, Mosqueda J, Garcia-Vazquez Z, Henke SE, George JE. Prevalence of infectious agents in free-ranging white-tailed deer in northeastern Mexico. Journal of Wildlife Diseases. 2008 Oct 1;44(4):1002-7.
127. Wolf KN, DePerno CS, Jenks JA, Stoskopf MK, Kennedy-Stoskopf S, Swanson CC, Brinkman TJ, Osborn RG, Tardiff JA. Selenium status and antibodies to selected pathogens in white-tailed deer (Odocoileus virginianus) in southern Minnesota. Journal of wildlife diseases. 2008 Jan 1;44(1):181-7.
128. Köppel C, Knopf L, Thür B, Vogt HR, Meli ML, Lutz H, Stärk KD. Bovine virus diarrhea and the vector-borne diseases Anaplasmosis and Bluetongue: a sero-surveillance in free-ranging red deer (Cervus elaphus) in selected areas of Switzerland. European journal of wildlife research. 2007 Aug;53:226-30.
129. Dubay SA, Noon TH, deVos Jr JC, Ockenfels RA. Serologic survey for pathogens potentially affecting pronghorn (Antilocapra americana) fawn recruitment in Arizona, USA. Journal of wildlife diseases. 2006 Oct 1;42(4):844-8.
130. Grøndahl C, Uttenthal Å, Houe H, Rasmussen TB, Hoyer MJ, Larsen LE. Characterisation of a pestivirus isolated from persistently infected mousedeer (Tragulus javanicus). Archives of Virology. 2003 Aug;148:1455-63.
131. Lillehaug A, Vikøren T, Larsen IL, Åkerstedt J, Tharaldsen J, Handeland K. Antibodies to ruminant alpha-herpesviruses and pestiviruses in Norwegian cervids. Journal of wildlife diseases. 2003 Oct 1;39(4):779-86.
132. Jones LR, Zandomeni R, Weber EL. Genetic typing of bovine viral diarrhea virus isolates from Argentina. Veterinary Microbiology. 2001 Aug 20;81(4):367-75.
133. Van Campen H, Ridpath J, Williams E, Cavender J, Edwards J, Smith S, Sawyer H. Isolation of bovine viral diarrhea virus from a free-ranging mule deer in Wyoming. Journal of Wildlife Diseases. 2001 Apr 1;37(2):306-11.
134. Nielsen LR, Houe H, Nielsen SS. Narrative review comparing principles and instruments used in three active surveillance and control programmes for Non-EU-regulated diseases in the Danish cattle population. Frontiers in Veterinary Science. 2021 Jul 19;8:685857.
135. Hause BM, Pillatzki A, Clement T, Bragg T, Ridpath J, Chase CC. Persistent infection of American bison (Bison bison) with bovine viral diarrhea virus and bosavirus. Veterinary Microbiology. 2021 Jan 1;252:108949.
136. Salwa A, Anusz K, Arent Z, Paprocka G, Kita J. Seroprevalence of selected viral and bacterial pathogens in free-ranging European bison. Pol. J. Vet. Sci. 2007;10:19-23.
137. Sausker EA, Dyer NW. Seroprevalence of OHV-2, BVDV, BHV-1, and BRSV in ranch-raised bison (Bison bison). Journal of veterinary diagnostic investigation. 2002 Jan;14(1):68-70.
138. Ma JG, Cong W, Zhang FH, Feng SY, Zhou DH, Wang YM, Zhu XQ, Yin H, Hu GX. Seroprevalence and risk factors of bovine viral diarrhoea virus (BVDV) infection in yaks (Bos grunniens) in northwest China. Tropical animal health and production. 2016 Dec;48:1747-50.
139. Van Campen H, Davis C, Flinchum JD, Bishop JV, Schiebel A, Duncan C, Spraker T. Epizootic hemorrhagic disease in yaks (Bos grunniens). Journal of Veterinary Diagnostic Investigation. 2013 May;25(3):443-6.
140. Mishra N, Rajukumar K, Vilcek S, Tiwari A, Satav JS, Dubey SC. Molecular characterization of bovine viral diarrhea virus type 2 isolate originating from a native Indian sheep (Ovies aries). Veterinary Microbiology. 2008 Jul 27;130(1-2):88-98.
141. Ataseven VS, Gürel K, Pestil Z, Ambarcıoğlu P, Doğan F, Kayhanlar M. BVDV, BHV-1 and BLV antibodies in dromedary camels of Turkey kept without and with ruminants. Tropical Animal Health and Production. 2022 Feb;54:1-6.
142. Liu Q, Liu L, Meng YK, Wang C, Gao Y, Zheng FG, Ma HL. Serological evidence of bovine viral diarrhea virus and peste des petits ruminants virus infection in alpacas (Vicugna pacos) in Shanxi Province, northern China. Tropical Animal Health and Production. 2021 May;53(2):299.
143. Dittmer KE, Hinkson JA, Dwyer C, Adlington B, van Andel M. Prevalence of Candidatus Mycoplasma haemolamae, bovine viral diarrhoea virus, and gastrointestinal parasitism in a sample of adult New Zealand alpaca (Vicugna pacos). New Zealand veterinary journal. 2018 Jan 2;66(1):9-15.
144. Saidi R, Bessas A, Bitam I, Ergün Y, Ataseven VS. Bovine herpesvirus-1 (BHV-1), bovine leukemia virus (BLV) and bovine viral diarrhea virus (BVDV) infections in Algerian dromedary camels (Camelus dromaderius). Tropical animal health and production. 2018 Mar;50:561-4.
145. Malek S, Madkour B. Detection of Antibodies to Bovine Viral Diarrhea Virus (BVDV) Disease in Imported Camels (Camelus dromedarius). Suez Canal Veterinary Medical Journal. SCVMJ. 2017 Dec 1;22(2):1-8.
146. Khalafalla AI, Al Eknah MM, Abdelaziz M, Ghoneim IM. A study on some reproductive disorders in dromedary camel herds in Saudi Arabia with special references to uterine infections and abortion. Tropical animal health and production. 2017 Jun;49:967-74.
147. Stanitznig A, Lambacher B, Eichinger M, Franz S, Wittek T. Prevalence of important viral infections in new world camelids in Austria. Wien. Tierärztl. Mon. 2016 Jan 1;103:92-100.
148. Al-Rubayie KM. Detection of bovine viral diarrhea-mucosal disease (BVDMD) virus in Dromedary camel in Iraq using ELISA/A preliminary study. Mirror Res. Vet. Sci. Anim. 2016;5(Special issue):70-4.
149. Peter ID, El-Yuguda AD, Mshelia GD, Dawurung JA. Detection of bovine viral diarrhea virus antibodies in camels (Camelus dromedarius) in Maiduguri, Nigeria. Sokoto Journal of Veterinary Sciences. 2015;13(3):49-52.
150. Gao S, Luo J, Du J, Lang Y, Cong G, Shao J, Lin T, Zhao F, Belák S, Liu L, Chang H. Serological and molecular evidence for natural infection of Bactrian camels with multiple subgenotypes of bovine viral diarrhea virus in Western China. Veterinary microbiology. 2013 Apr 12;163(1-2):172-6.
151. Raoofi A, Hemmatzadeh F, Ghanaei AM. Serological survey of antibodies against BVD virus in camels (Camelus dromedarius) in Iran. Tropical animal health and production. 2010 Mar;42:411-4.
152. Turcitu MA, Bărboi G, Vuță V, Zamfir L, Coste H, Boncea D. Preliminary results regarding bovine viral diarrhea virus epidemiology in Romanian wild boars. Analele Univ Spiru Haret. 2010;11.
153. Intisar KS, Ali YH, Khalafalla AI, Mahasin ER, Amin AS, Taha KM. The first report on the prevalence of pestivirus infection in camels in Sudan. Tropical animal health and production. 2010 Aug;42:1203-7.
154. Topliff CL, Smith DR, Clowser SL, Steffen DJ, Henningson JN, Brodersen BW, Bedenice D, Callan RJ, Reggiardo C, Kurth KL, Kelling CL. Prevalence of bovine viral diarrhea virus infections in alpacas in the United States. Journal of the American Veterinary Medical Association. 2009 Feb 15;234(4):519-29.
155. Foster AP, Houlihan M, Higgins RJ, Errington J, Ibata G, Wakeley PR. BVD virus in a British alpaca. The Veterinary Record. 2005 May 28;156(22):718.
156. Carman S, Carr N, DeLay J, Baxi M, Deregt D, Hazlett M. Bovine viral diarrhea virus in alpaca: abortion and persistent infection. Journal of Veterinary Diagnostic Investigation. 2005 Nov;17(6):589-93.
157. Wentz PA, Belknap EB, Brock KV, Collins JK, Pugh DG. Evaluation of bovine viral diarrhea virus in New World camelids. Journal of the American Veterinary Medical Association. 2003 Jul 15;223(2):223-8.
158. Goyal SM, Bouljihad M, Haugerud S, Ridpath JF. Isolation of bovine viral diarrhea virus from an alpaca. Journal of veterinary diagnostic investigation. 2002 Jan;14(6):523-5.
159. Belknap EB, Collins JK, Larsen RS, Conrad KP. Bovine viral diarrhea virus in New World camelids. Journal of Veterinary Diagnostic Investigation. 2000 Nov;12(6):568-70.
