# Supplementary material for: Molecular Epidemiology and Control Strategies for BVDV: A Global Systematic Review From 2000 to 2025
Source: Vet Med Int. 2025 Nov 12;2025:6732453. doi: 10.1155/vmi/6732453 (PMC12629698; doi:10.1155/vmi/6732453)
Supplement: Supporting Information 3 — Supporting Table 2: Different risk factors associated with BVDV infection in cattle herds documented in various countries. [file 6732453.f3.docx]

**Supplementary Table 2:** Different risk factors associated with BVDV infection in cattle herd documented in various countries.

| **Category** | **Identified risk factors** | **Country** | **References** |
| --- | --- | --- | --- |
| Animal related parameter | - Age of cattle - Previously aborted animals - History of repeated breeding - Multiparous cows - Congenital defective calve birth - History of respiratory disease - Native breed - Contact with pregnant cow - Lactation status - Species of animals - Weaning age - Fetal fluid | Egypt, Ethiopia, Turkey, Iran, Malaysia Indonesia, Colombia, Pakistan, China, French, Brazil, | [1-12] |
| Herd management practices | - Larger herd size - Farm size - Mixed farming - Intensive farming - Molasses supplementation - High animal density - Housing system | Nepal, Egypt, Ethiopia, Colombia, French, India, Brazil, Ecuador, Switzerland, Jordan  Pakistan, India, Ethiopia, Mexico, USA, | [1, 2, 6, 9, 12, 13, 14, 15, 16, 17] |
| Biosecurity practices | - Grazing on common pasture with small ruminant - Contact with wildlife animals - Movement of cattle for marketing - Purchase and introduction of new animals to herds - Grazing on alpine - Distance between farms - Movement of animals between herds - Exchange of animals, - Transfer of pregnant heifers into resident herds - Tools and equipment’ s that use on PI animals   contract farm workers | Turkey, Indonesia, Japan, Poland, Pakistan, Denmark, Finland, Iceland, Sweden, Switzerland, Norway, India, Japan, Brazil, Mexico, Ireland, USA, Jordan | [3, 5, 7, 12, 14, 16, 17, 18, 19, 20, 21, 22, 23, 24] |
| Breeding Managements | - Artificial insemination - Natural mating - Frozen semen - AI practitioner | Ethiopia, Turkey, Iran, Thailand, Brazil, | [3, 4, 12, 25] |
| Veterinarian and health practitioner | - Reuse of needle - Rectal palpation - Veterinary practitioners - Technicians | Iran, Colombia, Thailand, Ireland | [4, 6, 23, 25] |
| Environmental factors | - Geographical location, - Geographical origin of animal, - Untreated manure, - Season, - The altitude, - Distance between the manure pit and farm | Poland, Indonesia, French, India, China, Mexico, Ecuador | [5, 9, 11, 14, 15, 19, 22] |

**References**

1. Selim A, Marzok M, Abdelhady A, Gattan HS, Salem M, Al-Hammadi MA. Serosurvey and Associated Risk Factors for Bovine Viral Diarrhea Virus Infection in Dromedary Camels in Egypt. Transboundary and Emerging Diseases. 2024 Feb 10;2024.
2. Birhanu W, Tesfaye A, Getachew Y, Negussie H. Seroprevalence of bovine viral diarrhea virus and detection of persistently infected (PI) animals in dairy farms of Holeta, central Ethiopia. Ethiopian Veterinary Journal. 2024 Mar 18;28(1):73-87.
3. İnce ÖB, Ayaz A. Seroprevalence and risk factors associated with bovine viral diarrhoea virus in Turkey. Tropical Animal Health and Production. 2023 Aug;55(4):246.
4. Hashemi M, Bakhshesh M, Manavian M. Bovine viral diarrhea virus and bovine herpes virus-1 in dairy cattle herds in Fars province, Southern Iran: seroprevalence and evaluation of risk factors. Archives of Razi Institute. 2022 Oct;77(5):1621.
5. Nugroho W, Silitonga RJ, Reichel MP, Irianingsih SH, Wicaksono MS. The epidemiology and control of bovine viral diarrhoea virus in tropical Indonesian cattle. Pathogens. 2022 Feb 7;11(2):215.
6. Martínez-Rodríguez LC, Guzmán-Barragán BL, Ordoñez D, Tafur-Gómez GA. Cattle seroprevalence and risk factors associated with bovine viral diarrhea in the northeastern of Colombia. Tropical Animal Health and Production. 2021 Jul;53(3):377.
7. Raheem A, Ahmad A, Rabbani M, Ghafoor A, Ajnum AA, Avais M, Ramiz RM, Ur-Rehman H. Determination of sero-prevalence and associated risk factors of bovine viral diarrhea virus (BVDV) in bovine population from southern Punjab, Pakistan. The Journal of Animal & Plant Sciences, 30(3): 2020, Page: 545-551.
8. Ran X, Chen X, Ma L, Wen X, Zhai J, Wang M, Tong X, Hou G, Ni H. A systematic review and meta-analysis of the epidemiology of bovine viral diarrhea virus (BVDV) infection in dairy cattle in China. Acta tropica. 2019 Feb 1;190:296-303.
9. Qi L, Beaunée G, Arnoux S, Dutta BL, Joly A, Vergu E, Ezanno P. Neighbourhood contacts and trade movements drive the regional spread of bovine viral diarrhoea virus (BVDV). Veterinary research. 2019 Apr 29;50(1):30.
10. Daves L, Yimer N, Arshad SS, Sarsaifi K, Omar M, Yusoff R, Haron A, Abdullah F. Seroprevalence of bovine viral diarrhea virus (BVDV) infection and associated risk factors in cattle in Selangor, Malaysia. Vet. Med. Open J. 2016;1:22-8.
11. Ma JG, Cong W, Zhang FH, Feng SY, Zhou DH, Wang YM, Zhu XQ, Yin H, Hu GX. Seroprevalence and risk factors of bovine viral diarrhoea virus (BVDV) infection in yaks (Bos grunniens) in northwest China. Tropical animal health and production. 2016 Dec;48:1747-50.
12. Marques AL, de Oliveira Assis AC, Simões SV, de Lima Tolentino ML, de Azevedo SS. Risk factors associated with Bovine Viral Diarrhea Virus (BVDV) infection in the semiarid of the state of Paraíba, in the northeast region of Brazil. Semina: Ciências Agrárias. 2016;37(5):3095-105.
13. Gautam A, Dhakal S, Sharma U, Khanal D, Kaphle K. Seroprevalence and its associated risk factors of Bovine Neosporosis and Bovine Viral Diarrhea in cattle of Tilottama municipality, Rupandehi, Nepal. Int J Vet Sci Res. 2022;8(3):127-32.
14. Kumar SK, Palanivel KM, Sukumar K, Ronald BS, Selvaraju G, Ponnudurai G. Herd-level risk factors for bovine viral diarrhea infection in cattle of Tamil Nadu. Tropical animal health and production. 2018 Apr;50:793-9.
15. Saa LR, Perea A, García-Bocanegra I, Arenas AJ, Jara DV, Ramos R, Carbonero A. Seroprevalence and risk factors associated with bovine viral diarrhea virus (BVDV) infection in non-vaccinated dairy and dual purpose cattle herds in Ecuador. Tropical animal health and production. 2012 Mar;44:645-9.
16. Presi P, Struchen R, Knight-Jones T, Scholl S, Heim D. Bovine viral diarrhea (BVD) eradication in Switzerland—experiences of the first two years. Preventive veterinary medicine. 2011 May 1;99(2-4):112-21.
17. Talafha AQ, Hirche SM, Ababneh MM, Al-Majali AM, Ababneh MM. Prevalence and risk factors associated with bovine viral diarrhea virus infection in dairy herds in Jordan. Tropical animal health and production. 2009 Apr;41:499-506.
18. Nishimori A, Hirose S, Ogino S, Andoh K, Isoda N, Sakoda Y. Endemic infections of bovine viral diarrhea virus genotypes 1b and 2a isolated from cattle in Japan between 2014 and 2020. Journal of Veterinary Medical Science. 2022;84(2):228-32.
19. Rypuła K, Płoneczka-Janeczko K, Czopowicz M, Klimowicz-Bodys MD, Shabunin S, Siegwalt G. Occurrence of BVDV infection and the presence of potential risk factors in dairy cattle herds in Poland. Animals. 2020 Jan 31;10(2):230.
20. Moennig V, Becher P. Control of bovine viral diarrhea. Pathogens. 2018 Mar 8;7(1):29.
21. Sekiguchi S, Presi P, Omori R, Staerk K, Schuppers M, Isoda N, Yoshikawa Y, Umemura T, Nakayama H, Fujii Y, Sakoda Y. Evaluation of bovine viral diarrhoea virus control strategies in dairy herds in Hokkaido, Japan, using stochastic modelling. Transboundary and emerging diseases. 2018 Feb;65(1):e135-44.
22. Segura-Correa JC, Zapata-Campos CC, Jasso-Obregón JO, Martinez-Burnes J, López-Zavala R. Seroprevalence and risk factors associated with bovine herpesvirus 1 and bovine viral diarrhea virus in North-Eastern Mexico. Open veterinary journal. 2016 Aug 26;6(2):143-9.
23. Sayers RG, Byrne N, O'Doherty E, Arkins S. Prevalence of exposure to bovine viral diarrhoea virus (BVDV) and bovine herpesvirus-1 (BoHV-1) in Irish dairy herds. Research in Veterinary Science. 2015 Jun 1;100:21-30.
24. Ridpath J. Preventive strategy for BVDV infection in North America. Japanese Journal of Veterinary Research. 2012 Feb;60(Supplement):S41-9.
25. Nilnont T, Aiumlamai S, Kanistanont K, Inchaisri C, Kampa J. Bovine viral diarrhea virus (BVDV) infection in dairy cattle herds in northeast Thailand. Tropical animal health and production. 2016 Aug;48:1201-8.
