# Supplementary material for: Molecular Epidemiology and Control Strategies for BVDV: A Global Systematic Review From 2000 to 2025
Source: Vet Med Int. 2025 Nov 12;2025:6732453. doi: 10.1155/vmi/6732453 (PMC12629698; doi:10.1155/vmi/6732453)
Supplement: Supporting Information 4 — Supporting Table 3: Prevention and control strategies for BVDV implemented by different countries across the globe. [file 6732453.f4.docx]

**Supplementary table 3: Prevention and control strategies for BVDV and implemented by different countries across the globe.**

| **Study reference** | **Country** | **Prevention and control strategy for BVDV** | | | | | |
| --- | --- | --- | --- | --- | --- | --- | --- |
|  |  | **Vaccination** | | **Culling of PI animals** | **Movement control of infected animals** | **Strict Biosecurity** | **Routine surveillance**  **(Using test**) |
|  |  | **Live** | **Killed** |  |  |  |  |
| Selim et al., 2024 | Egypt | N/A | N/A | N/A | N/A | N/A | Ab-ELISA |
| Birhanu et al., 2024 | Ethiopia | N/A | N/A | N/A | N/A | N/A | Ab-ELISA |
| İnce et al., 2023 | Turkey | N/A | N/A | N/A | N/A | N/A | Ab-ELISA, RT-PCR |
| Fountain et al., 2022 | Australia | yes | N/A | N/A | N/A | yes | N/A |
| Hashemi et al., 2022 | Iran | N/A | N/A | N/A | N/A | N/A | Ab-ELISA, Ag-ELISA |
| Nugroho et al., 2022 | Indonesia | Modified live vaccines | N/A | yes | yes | yes | Ab-ELISA, Ag-ELISA |
| Nishimori et al., 2022 | Japan | Attenuated live vaccine | Inactivated vaccine | yes | yes | N/A | Ab-ELISA (From Bulk milk tank) |
| Prosser et al., 2022 | United Kingdom | yes | N/A | yes | yes | yes | Ab-ELISA, Ag-ELISA |
| Gautam et al., 2022 | Nepal | N/A | N/A | N/A | N/A | N/A | Ab-ELISA |
| Nielsen et al., 2021 | Denmark | yes | N/A | yes | N/A | yes | Ab-ELISA, Ag-ELISA |
| Gomez-Romero et al., 2021 | Mexico | Modified live (MLV) | Inactivated virus vaccines | N/A | N/A | yes | qRT-PCR, Ab-ELISA, VNT |
| Martínez-Rodríguez et al., 2021 | Colombia | N/A | N/A | N/A | N/A | N/A | Ab-ELISA |
| Rypuła et al.,2020 | Poland | N/A | N/A | N/A | N/A | N/A | Ab-ELISA |
| Long et al., 2020 | Australia | yes | N/A | yes | N/A | yes | Ab-ELISA, Ag-ELISA, RT-PCR |
| Han et al., 2020 | New Zealand | N/A | yes | yes | N/A | yes | Ab-ELISA, Ag-ELISA, RT-PCR |
| Raheem et al., 2020 | Pakistan | N/A | N/A | N/A | N/A | N/A | Ab-ELISA |
| Ran et al., 2019 | China | yes | N/A | yes | N/A | yes | RT-PCR, Ab-ELISA, Ag-ELISA |
| Qi et al., 2019 | French | N/A | N/A | yes | N/A | yes | - |
| Gates et al., 2019 | New Zealand | N/A | Inactivated  (Combined vaccine Bovilis BVDa, Ultravac BVDb ,Hiprabovis 3) | yes | N/A | yes | Ab-ELISA, Ag-ELISA, RT-PCR |
| Gethmann et al., 2019 | German | Modified live | yes | yes | yes | yes | Ab-ELISA, Ag-ELISA, RT-PCR |
| Moennig and Becher, 2018 | Austria | N/A | N/A | yes | yes | yes | Ab-ELISA, Ag-ELISA |
| Moennig and Becher, 2018 | Belgium | yes | N/A | yes | yes | N/A | - |
| Moennig and Becher, 2018 | Denmark | N/A | N/A | yes | yes | yes | Ab-ELISA, Ag-ELISA |
| Moennig and Becher, 2018 | Finland | N/A | N/A | yes | yes | yes | Ab-ELISA, Ag-ELISA |
| Moennig and Becher, 2018 | Germany | yes | N/A | yes | yes | N/A | - |
| Kumar et al., 2018 | India | N/A | N/A | N/A | N/A | N/A | Ab-ELISA |
| Moennig and Becher, 2018 | Germany | Modified live vaccine (MLV) | N/A | yes | yes | yes | Ab-ELISA, Ag-ELISA |
| Moennig and Becher, 2018 | Iceland | N/A | N/A | yes | yes | yes | Ab-ELISA, Ag-ELISA |
| Moennig and Becher, 2018 | Ireland | yes | N/A | yes | yes | N/A | - |
| Sekiguchi et al., 2018 | Japan | yes | N/A | yes | yes | N/A | RT-PCR, Ab- ELISA |
| Moennig and Becher, 2018 | Norway | N/A | N/A | yes | yes | yes | Ab-ELISA, Ag-ELISA |
| Moennig and Becher, 2018 | Sweden | N/A | N/A | yes | yes | yes | Ab-ELISA, Ag-ELISA |
| Aragaw et al., 2018 | Ethiopia | N/A | N/A | N/A | N/A | N/A | Ab-ELISA |
| Moennig and Becher, 2018 | Switzerland | (In 2013 vaccination against BVD was banned) | N/A | yes | yes | yes | Ab-ELISA, Ag-ELISA |
| Moennig and Becher, 2018 | Scotland | yes | N/A | yes | yes | N/A | - |
| Wernike et al., 2017 | Germany | yes | N/A | N/A | N/A | N/A | - |
| Nilnont et al., 2016 | Thailand | N/A | N/A | yes | N/A | N/A | - |
| Daves et al., 2016 | Malaysia | N/A | N/A | N/A | N/A | N/A | Ab-ELISA |
| Ma et al., 2016 | China | N/A | N/A | N/A | N/A | N/A | Ab-ELISA, Ag-ELISA |
| Marques et al., 2016 | Brazil | N/A | N/A | N/A | N/A | N/A | VNT, Ab-ELISA |
| Segura-Correa et al., 2016 | Mexico | N/A | N/A | N/A | N/A | N/A | Ab-ELISA |
| Sayers et al., 2015 | Ireland | Yes | N/A | N/A | N/A | N/A | Ab-ELISA (Bulk  milk antibody test) |
| Griebel, 2015 | United States | yes | Yes | N/A | N/A | N/A | - |
| Schirrmeier, 2014 | Germany | yes | N/A | yes | yes | yes | Ab-ELISA, Ag-ELISA, RT-PCR |
| Jarvinen et al., 2014 | United States | yes | N/A | yes | yes | yes | VNT |
| Løken and Nyberg, 2013 | Norway | yes | N/A | yes | yes | N/A | Ab-ELISA, Ag-ELISA, RT-PCR |
| Srinivas and Srikanth, 2012 | India | Modified live vaccine | N/A | yes | N/A | yes | - |
| Stott et al., 2012 | Ireland | yes | N/A | yes | N/A | N/A | Ab-ELISA, Ag-ELISA, RT-PCR |
| Saa et al., 2012 | Ecuador | N/A | N/A | N/A | N/A | N/A | Ab-ELISA |
| Häsler et al., 2012 | Switzerland | N/A | N/A | yes | yes | N/A | Ab-ELISA, Ag-ELISA |
| Ridpath, 2012 | United States | yes | N/A | yes | N/A | yes | RT-PCR |
| Presi et al., 2011 | Switzerland | N/A | N/A | yes | N/A | N/A | Ag-ELISA |
| Weldegebriel et al., 2009 | United Kingdom | N/A | Killed vaccine | yes | N/A | yes | - |
| Talafha et al., 2009 | Jordan | N/A | N/A | N/A | N/A | N/A | Ab-ELISA |
| Houe et al., 2006 | European countries | yes | N/A | yes | N/A | N/A | Ab-ELISA, Ag-ELISA, RT-PCR |
| Lindberg et al., 2005 | Europe countries | yes | N/A | yes | yes | yes | - |
| Valle et al., 2005 | Norway | N/A | N/A | yes | yes | N/A | Ab-ELISA, Ag-ELISA, RT-PCR |
| Moennig et al., 2005 | Germany | yes | yes | yes | yes | yes | Ab-ELISA, Ag-ELISA, RT-PCR |
| Sandvik et al., 2004 | Europe countries | yes | N/A | yes | N/A | yes | Ab-ELISA, Ag-ELISA, RT-PCR |
| Yasutomi et al., 2004 | Japan | yes | N/A | N/A | N/A | N/A | - |
| Chi et al., 2002 | Canada | yes | N/A | yes | N/A | yes | - |
| Fray et al., 2000 | Europe countries | N/A | yes | yes | N/A | yes | Ab-ELISA, Ag-ELISA, RT-PCR |

N/A: not applicable; Ag-ELISA: Antigen-capture enzyme linked immunosorbent assay; Ab-ELISA: Antibody-capture enzyme linked immunosorbent assay, RT-PCR: Reverse transcription polymerase chain reaction; RT-qPCR: Quantitative reverse transcription polymerase chain reaction; VNT: Virus neutralization test;

**References**

1. Selim A, Marzok M, Abdelhady A, Gattan HS, Salem M, Al-Hammadi MA. Serosurvey and Associated Risk Factors for Bovine Viral Diarrhea Virus Infection in Dromedary Camels in Egypt. Transboundary and Emerging Diseases. 2024 Feb 10;2024.
2. Birhanu W, Tesfaye A, Getachew Y, Negussie H. Seroprevalence of bovine viral diarrhea virus and detection of persistently infected (PI) animals in dairy farms of Holeta, central Ethiopia. Ethiopian Veterinary Journal. 2024 Mar 18;28(1):73-87.
3. İnce ÖB, Ayaz A. Seroprevalence and risk factors associated with bovine viral diarrhoea virus in Turkey. Tropical Animal Health and Production. 2023 Aug;55(4):246.
4. Fountain J, Brookes V, Kirkeby C, Manyweathers J, Maru Y, Hernandez-Jover M. One size does not fit all: Exploring the economic and non-economic outcomes of on-farm biosecurity for bovine viral diarrhoea virus in Australian beef production. Preventive Veterinary Medicine. 2022 Nov 1;208:105758.
5. Hashemi M, Bakhshesh M, Manavian M. Bovine viral diarrhea virus and bovine herpes virus-1 in dairy cattle herds in Fars province, Southern Iran: seroprevalence and evaluation of risk factors. Archives of Razi Institute. 2022 Oct;77(5):1621.
6. Nugroho W, Silitonga RJ, Reichel MP, Irianingsih SH, Wicaksono MS. The epidemiology and control of bovine viral diarrhoea virus in tropical Indonesian cattle. Pathogens. 2022 Feb 7;11(2):215.
7. Nishimori A, Hirose S, Ogino S, Andoh K, Isoda N, Sakoda Y. Endemic infections of bovine viral diarrhea virus genotypes 1b and 2a isolated from cattle in Japan between 2014 and 2020. Journal of Veterinary Medical Science. 2022;84(2):228-32.
8. Prosser NS, Green MJ, Ferguson E, Tildesley MJ, Hill EM, Keeling MJ, Kaler J. Cattle farmer psychosocial profiles and their association with control strategies for bovine viral diarrhea. Journal of Dairy Science. 2022 Apr 1;105(4):3559-73.
9. Gautam A, Dhakal S, Sharma U, Khanal D, Kaphle K. Seroprevalence and its associated risk factors of Bovine Neosporosis and Bovine Viral Diarrhea in cattle of Tilottama municipality, Rupandehi, Nepal. Int J Vet Sci Res. 2022;8(3):127-32.
10. Nielsen LR, Houe H, Nielsen SS. Narrative review comparing principles and instruments used in three active surveillance and control programmes for Non-EU-regulated diseases in the Danish cattle population. Frontiers in Veterinary Science. 2021 Jul 19;8:685857.
11. Gomez-Romero N, Ridpath JF, Basurto-Alcantara FJ, Verdugo-Rodriguez A. Bovine viral diarrhea virus in cattle from Mexico: Current Status. Frontiers in Veterinary Science. 2021 Aug 13;8:673577.
12. Martínez-Rodríguez LC, Guzmán-Barragán BL, Ordoñez D, Tafur-Gómez GA. Cattle seroprevalence and risk factors associated with bovine viral diarrhea in the northeastern of Colombia. Tropical Animal Health and Production. 2021 Jul;53(3):377.
13. Rypuła K, Płoneczka-Janeczko K, Czopowicz M, Klimowicz-Bodys MD, Shabunin S, Siegwalt G. Occurrence of BVDV infection and the presence of potential risk factors in dairy cattle herds in Poland. Animals. 2020 Jan 31;10(2):230.
14. Long R, Allworth MB, Smith AK, Hayes L, Hernandez‐Jover M. Knowledge, attitudes and management of bovine viral diarrhoea virus among eastern Australian cattle producers: results from a 2013 cross‐sectional study. Australian veterinary journal. 2020 Sep;98(9):429-37.
15. Han JH, Weston JF, Heuer C, Gates MC. Modelling the economics of bovine viral diarrhoea virus control in pastoral dairy and beef cattle herds. Preventive veterinary medicine. 2020 Sep 1;182:105092
16. Raheem A, Ahmad A, Rabbani M, Ghafoor A, Ajnum AA, Avais M, Ramiz RM, Ur-Rehman H. Determination of sero-prevalence and associated risk factors of bovine viral diarrhea virus (BVDV) in bovine population from southern Punjab, Pakistan. The Journal of Animal & Plant Sciences, 30(3): 2020, Page: 545-551.
17. Ran X, Chen X, Ma L, Wen X, Zhai J, Wang M, Tong X, Hou G, Ni H. A systematic review and meta-analysis of the epidemiology of bovine viral diarrhea virus (BVDV) infection in dairy cattle in China. Acta tropica. 2019 Feb 1;190:296-303.
18. Qi L, Beaunée G, Arnoux S, Dutta BL, Joly A, Vergu E, Ezanno P. Neighbourhood contacts and trade movements drive the regional spread of bovine viral diarrhoea virus (BVDV). Veterinary research. 2019 Apr 29;50(1):30.
19. Gates MC, Evans CA, Weir AM, Heuer C, Weston JF. Recommendations for the testing and control of bovine viral diarrhoea in New Zealand pastoral cattle production systems. New Zealand veterinary journal. 2019 Sep 3;67(5):219-27.
20. Gethmann J, Probst C, Bassett J, Blunk P, Hövel P, Conraths FJ. An epidemiological and economic simulation model to evaluate strategies for the control of bovine virus diarrhea in Germany. Frontiers in Veterinary Science. 2019 Nov 19;6:406.
21. Moennig V, Becher P. Control of bovine viral diarrhea. Pathogens. 2018 Mar 8;7(1):29.
22. Kumar SK, Palanivel KM, Sukumar K, Ronald BS, Selvaraju G, Ponnudurai G. Herd-level risk factors for bovine viral diarrhea infection in cattle of Tamil Nadu. Tropical animal health and production. 2018 Apr;50:793-9.
23. Sekiguchi S, Presi P, Omori R, Staerk K, Schuppers M, Isoda N, Yoshikawa Y, Umemura T, Nakayama H, Fujii Y, Sakoda Y. Evaluation of bovine viral diarrhoea virus control strategies in dairy herds in Hokkaido, Japan, using stochastic modelling. Transboundary and emerging diseases. 2018 Feb;65(1):e135-44.
24. Aragaw K, Sibhat B, Ayelet G, Skjerve E, Gebremedhin EZ, Asmare K. Seroprevalence and factors associated with bovine viral diarrhea virus (BVDV) infection in dairy cattle in three milksheds in Ethiopia. Tropical animal health and production. 2018 Dec;50:1821-7.
25. Wernike K, Schirrmeier H, Strebelow HG, Beer M. Eradication of bovine viral diarrhea virus in Germany—Diversity of subtypes and detection of live-vaccine viruses. Veterinary microbiology. 2017 Sep 1;208:25-9.
26. Nilnont T, Aiumlamai S, Kanistanont K, Inchaisri C, Kampa J. Bovine viral diarrhea virus (BVDV) infection in dairy cattle herds in northeast Thailand. Tropical animal health and production. 2016 Aug;48:1201-8.
27. Daves L, Yimer N, Arshad SS, Sarsaifi K, Omar M, Yusoff R, Haron A, Abdullah F. Seroprevalence of bovine viral diarrhea virus (BVDV) infection and associated risk factors in cattle in Selangor, Malaysia. Vet. Med. Open J. 2016;1:22-8.
28. Ma JG, Cong W, Zhang FH, Feng SY, Zhou DH, Wang YM, Zhu XQ, Yin H, Hu GX. Seroprevalence and risk factors of bovine viral diarrhoea virus (BVDV) infection in yaks (Bos grunniens) in northwest China. Tropical animal health and production. 2016 Dec;48:1747-50.
29. Marques AL, de Oliveira Assis AC, Simões SV, de Lima Tolentino ML, de Azevedo SS. Risk factors associated with Bovine Viral Diarrhea Virus (BVDV) infection in the semiarid of the state of Paraíba, in the northeast region of Brazil. Semina: Ciências Agrárias. 2016;37(5):3095-105.
30. Segura-Correa JC, Zapata-Campos CC, Jasso-Obregón JO, Martinez-Burnes J, López-Zavala R. Seroprevalence and risk factors associated with bovine herpesvirus 1 and bovine viral diarrhea virus in North-Eastern Mexico. Open veterinary journal. 2016 Aug 26;6(2):143-9.
31. Sayers RG, Byrne N, O'Doherty E, Arkins S. Prevalence of exposure to bovine viral diarrhoea virus (BVDV) and bovine herpesvirus-1 (BoHV-1) in Irish dairy herds. Research in Veterinary Science. 2015 Jun 1;100:21-30.
32. Griebel PJ. BVDV vaccination in North America: risks versus benefits. Animal health research reviews. 2015 Jun;16(1):27-32.
33. Schirrmeier H. Three years of mandatory BVD control in Germany–lessons to be learned. InProceedings of the XXVIII World Buiatrics Congress, Cairns 2014 Jul 27.
34. Jarvinen JA, O'Connor AM. Seroprevalence of bovine viral diarrhea virus in alpacas in the United States and assessment of risk factors for exposure, 2006–2007. Journal of the American Veterinary Medical Association. 2014 Sep 15;245(6):696-703.
35. Løken T, Nyberg O. Eradication of BVDV in cattle: the Norwegian project. Veterinary Record. 2013 Jun;172(25):661-.
36. Srinivas M, Srikanth NR. Identification and control strategies of bovine viral diarrhoea virus (BVDV). International Journal of Pharma and Bio Sciences. 2012;3(4).
37. Stott AW, Humphry RW, Gunn GJ, Higgins I, Hennessy T, O’Flaherty J, Graham DA. Predicted costs and benefits of eradicating BVDV from Ireland. Irish veterinary journal. 2012 Dec;65(1):1-1.
38. Saa LR, Perea A, García-Bocanegra I, Arenas AJ, Jara DV, Ramos R, Carbonero A. Seroprevalence and risk factors associated with bovine viral diarrhea virus (BVDV) infection in non-vaccinated dairy and dual purpose cattle herds in Ecuador. Tropical animal health and production. 2012 Mar;44:645-9.
39. Häsler B, Howe KS, Presi P, Stärk KD. An economic model to evaluate the mitigation programme for bovine viral diarrhoea in Switzerland. Preventive veterinary medicine. 2012 Sep 15;106(2):162-73.
40. Ridpath J. Preventive strategy for BVDV infection in North America. Japanese Journal of Veterinary Research. 2012 Feb;60(Supplement):S41-9.
41. Presi P, Struchen R, Knight-Jones T, Scholl S, Heim D. Bovine viral diarrhea (BVD) eradication in Switzerland—experiences of the first two years. Preventive veterinary medicine. 2011 May 1;99(2-4):112-21.
42. Weldegebriel HT, Gunn GJ, Stott AW. Evaluation of producer and consumer benefits resulting from eradication of bovine viral diarrhoea (BVD) in Scotland, United Kingdom. Preventive Veterinary Medicine. 2009 Jan 1;88(1):49-56.
43. Talafha AQ, Hirche SM, Ababneh MM, Al-Majali AM, Ababneh MM. Prevalence and risk factors associated with bovine viral diarrhea virus infection in dairy herds in Jordan. Tropical animal health and production. 2009 Apr;41:499-506.
44. Houe H, Lindberg A, Moennig V. Test strategies in bovine viral diarrhea virus control and eradication campaigns in Europe. Journal of Veterinary Diagnostic Investigation. 2006 Sep;18(5):427-36.
45. Lindberg A, Houe H. Characteristics in the epidemiology of bovine viral diarrhea virus (BVDV) of relevance to control. Preventive veterinary medicine. 2005 Nov 15;72(1-2):55-73.
46. Moennig V, Houe H, Lindberg A. BVD control in Europe: current status and perspectives. Animal Health Research Reviews. 2005 Jun;6(1):63-74
47. Sandvik T. Progress of control and prevention programs for bovine viral diarrhea virus in Europe. Veterinary Clinics: Food Animal Practice. 2004 Mar 1;20(1):151-69.
48. YASUTOMI I, OKAZAWA M, HARA Y. Epidemiological investigation of Bovine Viral Diarrhea Virus infection with evaluating risk factors. Journal of Veterinary Epidemiology. 2004 Dec 20;8(2):77-83.
49. Chi J, Weersink A, VanLeeuwen JA, Keefe GP. The economics of controlling infectious diseases on dairy farms. Canadian Journal of Agricultural Economics/Revue canadienne d'agroeconomie. 2002 Nov;50(3):237-56.
50. Fray MD, Paton DJ, Alenius S. The effects of bovine viral diarrhoea virus on cattle reproduction in relation to disease control. Animal Reproduction Science. 2000 Jul 2;60:615-27.
51. Valle PS, Skjerve E, Martin SW, Larssen RB, Østerås O, Nyberg O. Ten years of bovine virus diarrhoea virus (BVDV) control in Norway: a cost-benefit analysis. Preventive Veterinary Medicine. 2005 Nov 15;72(1-2):189-207.
